# Parallel-stacked aromatic molecules in hydrogen-bonded inorganic frameworks

**DOI:** 10.1038/s41467-021-27324-2

**Published:** 2021-12-10

**Authors:** Masayasu Igarashi, Takeshi Nozawa, Tomohiro Matsumoto, Fujio Yagihashi, Takashi Kikuchi, Kazuhiko Sato

**Affiliations:** 1 Interdisciplinary Research Center for Catalytic Chemistry, National Institute of Advanced Industrial Science and Technology (AIST), Tsukuba Central 5, 1-1-1 Higashi, Tsukuba, 305-8565 Japan; 2 Rigaku Corporation, 3-9-12 Matsubara-cho, Akishima-shi, Tokyo, 196-8666 Japan

**Keywords:** Self-assembly, Organometallic chemistry, Crystal engineering

## Abstract

By precisely constructing molecules and assembling these into well-defined supramolecular structures, novel physical properties and functionalities can be realized, and new areas of the chemical space can be accessed. In both materials science and biology, a deeper understanding of the properties and exploitation of the reversible character of weak bonds and interactions, such as hydrogen bonds and π–π interactions, is anticipated to lead to the development of materials with novel properties and functionalities. We apply the hydrogen-bonded organic frameworks (HOFs) strategy to inorganic materials science using the cubic octamer of orthosilicic acid, [Si_8_O_12_][OH]_8_, as a building block, and find that various types of hydrogen-bonded inorganic frameworks (HIFs). We succeed in parallel π-stacking pure benzene, thiophene, selenophene, *p*-benzoquinone, thiophene·*p*-benzoquinone, and benzene·*p*-benzoquinone polymers infinitely. These polymers interact via their π-systems by taking advantage of the flexible pores of the three-dimensional nano-honeycomb HIFs, which consist of periodic wide and narrow segments.

## Abstract (web summary)

Hydrogen-bonded inorganic frameworks are porous structures that may lead to novel materials with unprecedented properties and functionalities. Here the authors report the solid-state structures of orthosilicic acid-based hydrogen-bonded inorganic frameworks that can encapsulate small unsaturated cyclic molecules such as benzene, which are found stacked in parallel.

## Introduction

By precisely constructing molecules and assembling well-defined supramolecular structures, various novel physical properties and functionalities can be developed, and new research fields can be opened. In recent years, the preparation, structural analysis, physical properties, and applications of structurally well-defined frameworks that contain one or more of various kinds of intermolecular interactions, such as coordination bonds, covalent bonds, hydrogen bonds, and π–π interactions, have been intensively investigated. The most representative research area in this field is metal–organic frameworks (MOFs)^1,2^, which form networks by coordination bonds and can be used to construct well-defined structures such as zero-dimensional nanodots, one-dimensional nanowires, two-dimensional nanolayers, and three-dimensional porous coordination polymers (PCPs)^3^. Among these structures, PCPs have attracted particular attention for their applications in, e.g., gas storage and separation, catalysis, reaction fields, and sensors. Covalent organic frameworks (COFs)^1,4^, which form networks via strong covalent bonds, have also been actively investigated due to their chemical and thermal stability. Their two-dimensional covalently bonded frameworks assemble to form three-dimensional porous or mesh frameworks via π–π interactions between the π electrons of the unsaturated compounds incorporated in the frame. In these systems, π–π interactions simultaneously contribute to the construction of structurally highly controlled molecules despite their weak nature. Similarly, hydrogen bonds, which are reversible due to their weak bond energy, can also be used for the assembly of higher-order structures and to induce dynamic functionalities in molecules. In biological systems, hydrogen bonds and π–π interactions play important roles in forming structurally well-defined molecules and in the context of molecular recognition; the most familiar examples are the structure and functionality of deoxyribonucleic acids (DNAs) and enzymes. Moreover, owing to the characteristic flexibility of the hydrogen bond, as demonstrated in ice crystals, various types of hydrogen-bonded networks can easily be assembled depending on the conditions under which the network is grown.

In materials science and biology, a deeper understanding of the properties and exploitation of the reversible character of weak bonds and interactions, such as hydrogen bonds and π–π interactions, is anticipated to lead to the development of materials with novel properties and functionalities. Thus, hydrogen-bonded organic frameworks (HOFs)^5–11^ have also received increasing attention and are expected to find applications in a wide range of fields, as hydrogen-bonding is an effective way to control and tune their structures, as described above. Silanol compounds that possess multiple SiOH groups are effective building units for hydrogen-bonded supramolecular networks owing to their strong hydrogen-bonding ability^12–18^. In materials science, silanols play important roles as intermediates in the sol–gel process to produce organosilicon materials (e.g., silicone oils, rubbers, and resins), inorganic materials (e.g., silicas and zeolites), and organic–inorganic hybrid materials (e.g., silane coupling agents and silsesquioxane derivatives), because the SiOH groups can potentially be converted into siloxane bonds (Si–O–Si) using, e.g., heating, dehydration reactions, or catalytic reactions. Among the silanol species, orthosilicic acid (Si(OH)_4_)^19,20^, which consists of four hydroxy groups on a silicon atom, is the smallest inorganic silanol and the smallest building block for silicas and silicates, which are the most abundant natural substances in the earth’s crust.

Recently, we have isolated orthosilicic acid and its dimer, cyclic trimer, cyclic tetramer, and cubic octamer [Si_8_O_12_][OH]_8_ (Q_8_H_8_, **1**), which is regarded as a cubic silica sub-nano particle and known as the double-four-ring motif, and analyzed these by single-crystal X-ray diffraction (XRD) analyses^21,22^. We found that the cyclic trimer forms a hydrogen-bonded inorganic framework (HIF) with a one-dimensional nanotape morphology through hydrogen bonds in the crystal. Inspired by these hydrogen-bonded networks, we envisioned that relatively stable cubic octamer **1** with eight SiOH groups could potentially be suitable for constructing higher-order HIFs.

In this paper, we report that HIFs with one-dimensional nanochain, nanowire, and nanorod, two-dimensional nanosheet and nanomesh-sheet, and three-dimensional nano-honeycomb networks can be formed from silanol **1** as the sole building block by combining various co-crystallization solvents and carrying out recrystallization under various conditions. The solid-state structures of these compounds are unambiguously determined by single-crystal XRD studies. Most importantly, we report that various small unsaturated cyclic molecules such as benzene stack in parallel to infinity in the flexible nano-honeycomb pores of the three-dimensional nanostructures.

## Results

### Preparation and structural analysis of one- and two-dimensional HIFs

**Fig. 1:**
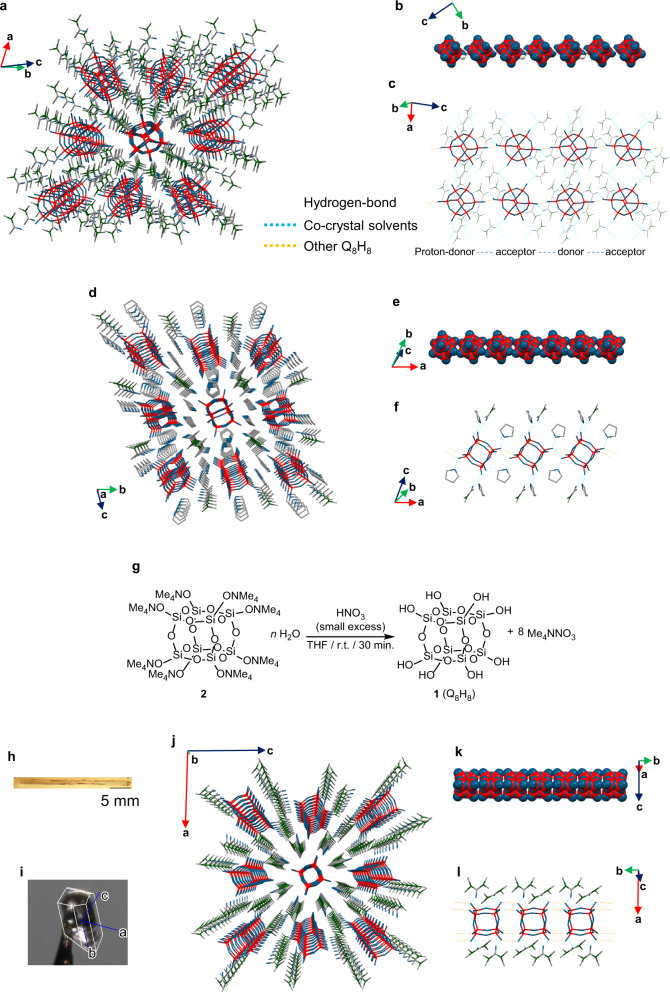
Crystal structures of 1D crystals. Crystal structures of 1D-C (**a**–**c**), 1D-W (**d**–**f**), and 1D-R (**h**–**l**) determined by single-crystal X-ray diffraction. The hydrogen atoms have been omitted for clarity, except for those involved in hydrogen-bonding between Q_8_H_8_ molecules in (**b** and **c**) (Si: red; O: blue; C: gray; H: white; N: green; hydrogen bonds between Q_8_H_8_: orange dashed lines; hydrogen bonds between Q_8_H_8_ and co-crystallization solvents: light blue dashed lines). **a**, **d**, **j** Crystal packing; **b**, **e**, **k** 3D space-filling representation of HIF’s network; **c**, **f**, **l** hydrogen-bonding interactions; **h** polarized micrograph; **i** crystal photograph with cell axes, which were drawn by CrysAlisPro software. **g** Scheme of optimized synthetic process of **1**.

We prepared and isolated the zero-dimensional cubic octamer [Si_8_O_12_][OH]_8_·10DMAc (**1**·10DMAc, 0D) by proton–ammonium exchange of the corresponding ammonium silicate [Si_8_O_20_][NMe_4_]_8_·*n*H_2_O (**2**) with 2,2-dimethyl-1,3-dioxane-4,6-dione (Meldrum’s acid), as reported in our previous work^22^. Purification by gel permeation chromatography (GPC), followed by recrystallization from a mixture of *N,N*-dimethylacetamide (DMAc) and diethyl ether (Et_2_O) afforded single crystals of **1** surrounded by ten DMAc molecules, which inhibited intermolecular interactions between molecules of **1**. The exchange of the co-crystallization solvent was examined by dissolving 0D crystals in other solvents for recrystallization. 0D crystals containing ten DMAc molecules were dissolved in diethyleneglycoldimethylether (diglyme) until saturation was reached and recrystallized at 4 °C; contrary to expectations, crystals incorporating no diglyme but five DMAc molecules per **1** molecule as a crystal solvent were obtained (**1**·5DMAc or [Si_8_O_12_][OH]_8_·5DMAc (1D-C)). The crystal structure of 1D-C was determined by single-crystal XRD analysis and is shown in Fig. 1a–c. Unexpectedly, not only the co-crystallization solvent (DMAc) but also the Si–OH groups at the diagonal vertices of **1** form hydrogen-bond networks with other molecules of **1** to form a chain-type HIF (Fig. 1b). Focusing on the hydrogen-bonding mode, two crystallographically independent molecules of **1** are arranged alternately in a chain with an alternating proton donor–acceptor relationship (Fig. 1c). On the other hand, when a single crystal of 0D was redissolved in tetrahydrofuran (THF) and recrystallized at −30 °C, one-dimensional HIF nanowire crystals (**1**·2DMAc·4THF or [Si_8_O_12_][OH]_8_·2DMAc·4THF (1D-W)), which contain a 1D chain of diagonally linked molecules of **1**, was obtained (Fig. 1d–f). The crystal contained four molecules of THF and two molecules of DMAc as co-crystallization solvents; the solvent molecules completely surround 1D-W through hydrogen bonds, thus separating each of the nanowires (Fig. 1d, f).

Despite the fact that the amount of DMAc in solution was much smaller than the amount of THF, two DMAc molecules remained coordinated to **1** due to their strong propensity to engage in hydrogen-bonding. Although DMAc is very effective in stabilizing such highly self-condensable silanols by inhibiting intermolecular interactions, its boiling point and toxicity are relatively high, i.e., it is desirable to avoid its use. The purification process using GPC and the high cost of Meldrum’s acid were also points for potential improvement in the synthesis. Thus, we investigated and optimized the reaction conditions carefully in order to develop a more convenient synthetic route. By using THF instead of DMAc, **1** could be isolated readily by the reaction of **2** with a variety of acids, such as nitric acid, hydrochloric acid, or sulfuric acid, and GPC purification was not required (Fig. 1g; for details of the reaction conditions, see the Supplementary information and Supplementary Fig. 1). Although Q_8_H_8_ is less stable in THF than in DMAc, it can be handled with little condensation by treating immediately after the reaction.

Having isolated a THF solution of **1**, we were then able to exchange THF for various solvents due to the relatively low boiling point of THF compared to that of DMAc and to recrystallize **1** under various conditions. We succeeded in obtaining single crystals of another one-dimensional HIF of **1** (**1**·4TMU or [Si_8_O_12_][OH]_8_·4TMU (1D-R)) with a rod-shaped geometry by recrystallizing **1** from a tetramethylurea (TMU) solution after exchanging the solvent from THF to TMU and then diffusing Et_2_O vapor into the solution as a poor solvent. Interestingly, the thus obtained crystals extended to approximately 2.5 cm in length within 2 days (Fig. 1h). The crystal structure of 1D-R was determined by single-crystal XRD analysis (Fig. 1j–l), which revealed that the long side of the crystal coincides with the *b*-axis of the unit cell and that the HIF is formed in the direction of the *b*-axis (Fig. 1i, j). Four molecules of TMU per molecule of **1** were present as the co-crystallization solvent. In the crystal, the hydrogen bonds are formed in a stacked manner with the cubic faces aligned along the *b*-axis. Figure 1k shows part of the infinite rod structure, in which each molecule of **1** is connected to two adjacent molecules of **1** through hydrogen bonds. Each molecule of **1** is further connected to four molecules of TMU through hydrogen bonds (Fig. 1l).

**Fig. 2:**
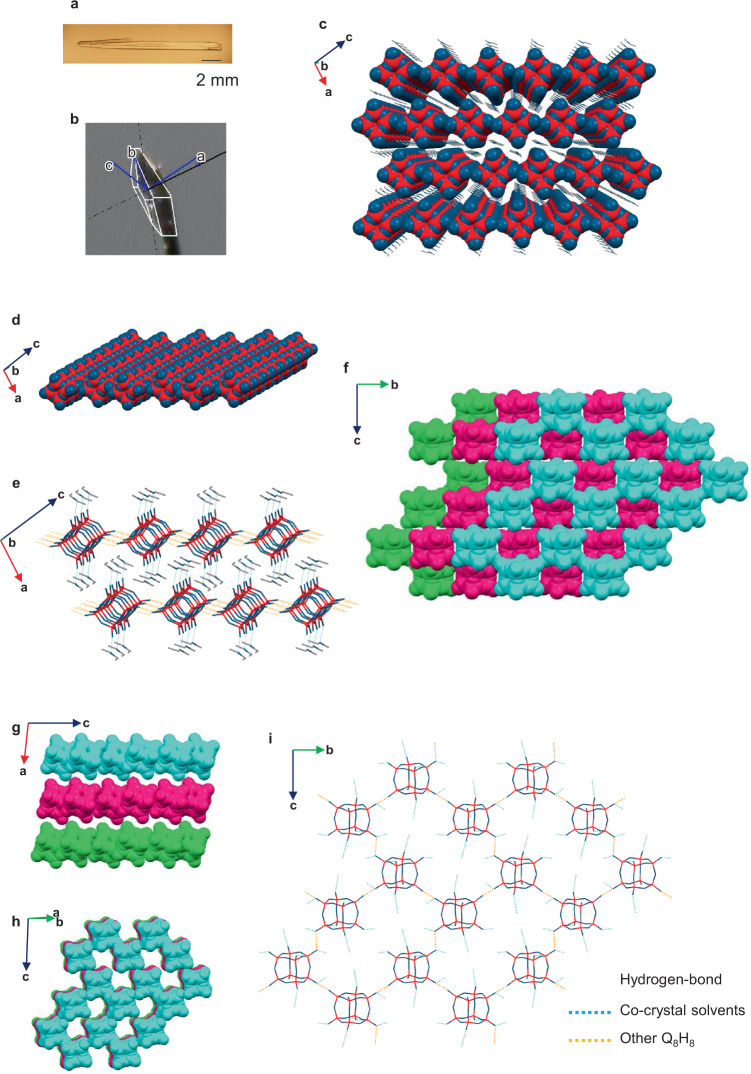
Crystal structures of 2D crystals. Crystal structures of 2D-S (**a**–**e**), and 2D-MS (**f**–**i**) determined by single-crystal X-ray diffraction. The hydrogen atoms have been omitted for clarity (Si: red; O: blue; C: gray; hydrogen bonds between Q_8_H_8_: orange dashed lines; hydrogen bonds between Q_8_H_8_ and co-crystallization solvents: light blue dashed lines). **a** Polarized micrograph; **b** crystal photograph with cell axes, which were drawn by CrysAlisPro software; **c** crystal packing; **d** 3D space-filling representation of HIF’s network; **e**, **i** hydrogen-bonding interactions; **f** top, **g** side, and **h** oblique space-filling views of three layers of the stacked mesh sheets of 2D-MS (co-solvent molecules omitted for clarity), in which the first, second, and third layers are colored light blue, pink, and green, respectively.

Diffusing Et_2_O vapor into a diglyme solution of **1** produced a framework in which the hydrogen-bonding networks extended indefinitely in two dimensions (**1**·2(Et_2_O) or [Si_8_O_12_][OH]_8_·2Et_2_O (2D-S)). The 2D-S crystals grew as plates, some of which reached approximately 1.5 cm in length within several days, and the two-dimensional HIF sheets were formed along the flat surface of the crystal (Fig. 2a, b). A single-crystal XRD analysis revealed that the two-dimensional sheet-type structure consists of one-dimensional rods aligned on a plane, with a displacement of half a molecule between sheets (Fig. 2c, d). Two co-crystallized Et_2_O molecules per molecule of **1** occupied the space between the two-dimensional nanolayers, with which they formed hydrogen bonds (Fig. 2e). Unexpectedly, crystals of another two-dimensional mesh-sheet-type network (**1**·6(DMAc) or [Si_8_O_12_][OH]_8_·6DMAc (2D-MS)) were obtained by preparing a diglyme solution of 0D at 45 °C and allowing it to stand at −18 °C (Fig. 2f–i). The mesh-sheet structure consists of a hydrogen-bonded network of rings that comprise six molecules of **1** (Fig. 2f, i). The two-dimensional nanolayers are stacked with a displacement of one molecule of **1** in the direction of the *b*-axis (Fig. 2f, g). Therefore, when the nanolayers were viewed obliquely, a cylindrical channel was observed (Fig. 2h).

### Preparation of three-dimensional HIF (3D⊃THF) and hydrogen-bonding manner of 1

Three-dimensionally hydrogen-bonded nano-honeycomb crystals (3D) of **1**·*n*THF (3D⊃THF) were obtained by simply evaporating the solvent from the THF solution of **1** at 30 °C (Supplementary Fig. 2). The three-dimensional nano-honeycomb HIF is constructed by expanding the hydrogen-bonding network of the two-dimensional HIF sheets displaced by half a molecule. Unfortunately, the location of the THF molecules, which were expected to be present in the nano-honeycomb pores, could not be confirmed clearly by the XRD analysis on account of the considerable disorder of the THF molecules, but the existence of the THF molecules was observed via nuclear magnetic resonance (NMR) spectroscopy of 3D⊃THF redissolved in DMF-*d*_6_ (Supplementary Fig. 2).

**Fig. 3:**
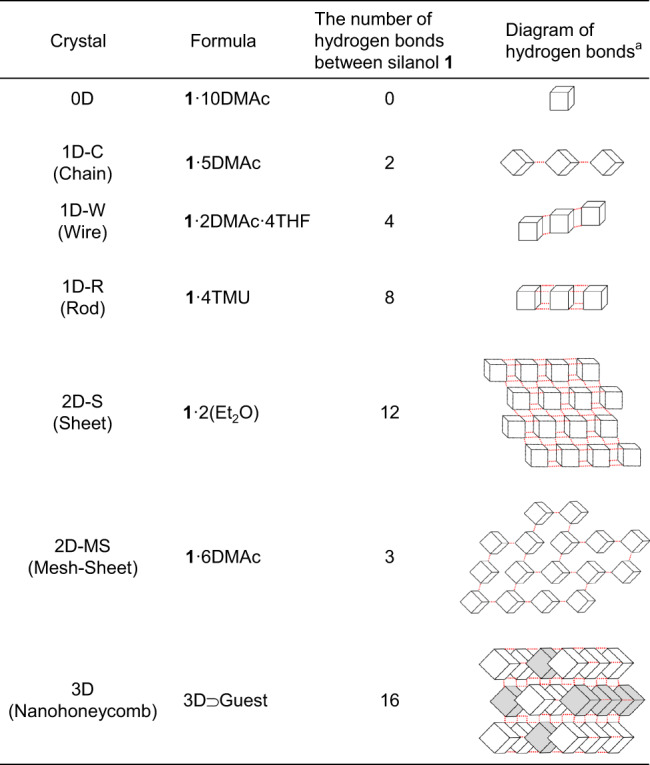
The summary of the hydrogen-bonding manner of [Si_8_O_12_][OH]_8_ (1). ^a^Cubes and red dashed lines indicate molecules of **1** and hydrogen bonds between silanol groups of **1**, respectively.

The type of crystal obtained seems to depend largely on the concentration of the co-crystallization solvent and the strength of its hydrogen-bonding ability toward **1**, i.e., the polarity. In other words, the number of hydrogen bonds formed by each molecule of **1** with other molecules of **1** to construct the HIF depends on the hydrogen-bonding ability of the crystal solvent and its concentration. Each molecule of **1** contains eight SiOH groups that can engage in the formation of hydrogen bonds, and each group can act as both a proton donor and acceptor; thus, a molecule of **1** can theoretically form up to 16 hydrogen bonds. As shown in Fig. 3, the number of hydrogen bonds per molecule of **1** in a HIF tends to be small when a recrystallization solvent with a high hydrogen-bonding ability (e.g., DMAc) is used, as this prevents the formation of hydrogen-bond networks among the molecules of **1**. In fact, when DMAc was incorporated as a co-crystallization solvent, lower concentrations of DMAc corresponded to higher numbers of hydrogen bonds, with each molecule of **1** forming zero hydrogen bonds with other molecules of **1** in 0D, two in 1D-C, and three in 2D-MS. On the other hand, if the hydrogen-bonding ability of the solvent is intermediate (e.g., TMU), the number of hydrogen bonds between molecules of **1** will be intermediate. In the case of TMU, which has a slightly lower hydrogen-bonding ability than DMAc, the networking number rises to eight in 1D-R. This number increases to 12 in 2D-S in the case of Et_2_O, which has a still lower hydrogen-bonding ability. Finally, when the solvent is evaporated from a THF solution of **1**, the maximum number of 16 in 3D will be obtained, with all SiOH groups participating in the formation of the HIF; the expansion of the hydrogen-bonding network among molecules of **1** dominates over the coordination of the co-crystallization solvent when solvents of lower hydrogen-bonding-ability are used (e.g., THF). As described above, even if the co-crystallization solvent is the same, three different types of crystals (0D, 1D-C, and 2D-MS) can be obtained from solutions with different DMAc concentrations; thus, the growth of the crystal depends also on the recrystallization process. Furthermore, the structure of these crystals appears to resemble the so-called microphase-separation phenomenon in polymer science^23^. The 0D and 2D crystals can be seen as spherical and lamellar structures, respectively. The 1D and 3D crystals have a rod-shaped structure and exhibit sea–island and phase-inverted morphology, respectively, with regards to **1** and the co-crystallization solvent.

### Preparation and structural analysis of three-dimensional **1**·2C_6_H_6_ or 1·2benzene (3D⊃benzene) crystals

The width of the nano-honeycomb-shaped pores of 3D⊃THF surrounded by the four rods of **1** is not uniform. The cross-section perpendicular to the direction of the long axis of the pore alternates periodically between wide and narrow sections (Supplementary Fig. 2). The period of the alternation (3.8 Å) was calculated as half the length of the cell in direction of the *c*-axis. Using a probe radius of 1.2 Å, the diameters of the narrow and wide sections of the solvent-accessible surface were estimated to be approximately 4 and 6 Å, respectively. Density functional theory (DFT) calculations for the structural optimization of a THF molecule were carried out at the B3LYP/6-31G(d) level of theory using the Gaussian 09 program^24^ and the distance between the most distant hydrogen atoms was calculated to be 4.19 Å. This width was almost the same as that of the narrow section of the pore; thus, the THF molecules did not exist in a specific position within the pore and their orientation and position could not be confirmed by X-ray crystallography. We thought that the periodic wide and narrow spaces could be useful for the precise alignment of molecules; in particular, we envisioned that small planar molecules larger than 4 Å and smaller than 6 Å would be confined in the wide sections and stacked infinitely at precisely the right separation to enable π–π interactions among small aromatic and unsaturated cyclic molecules. To test this hypothesis, we first attempted to obtain a parallel π–π stacked benzene polymer.

**Fig. 4:**
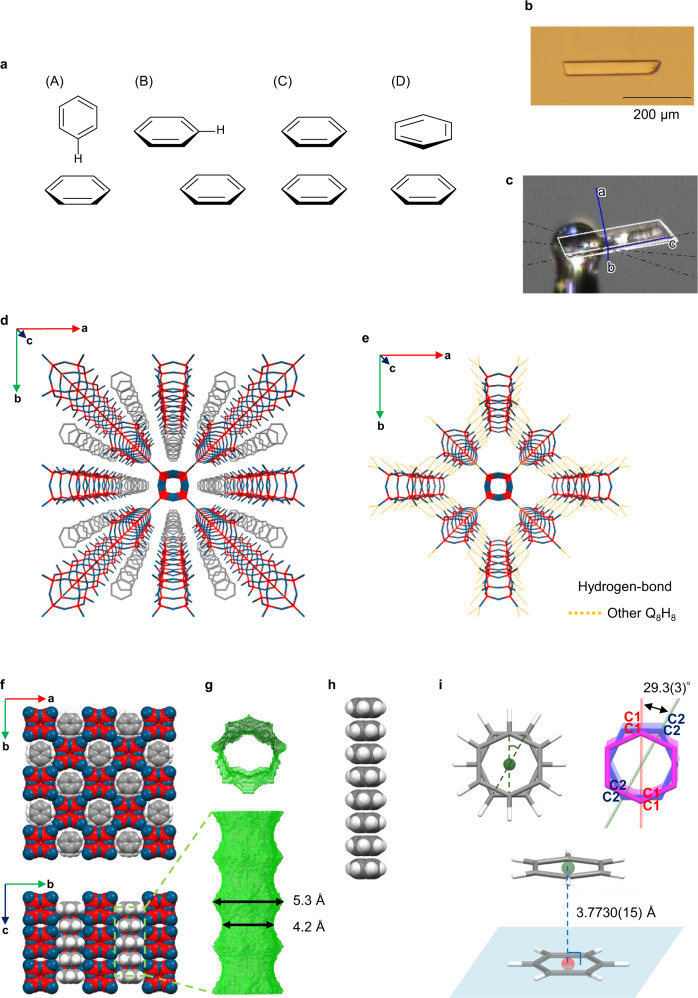
Geometries of benzene dimers and crystal structures of 3D⊃benzene determined by single-crystal X-ray diffraction. The hydrogen atoms in the 3D crystal packing representations have been omitted for clarity. Color code: Si = red; O = blue; C = gray; H = white. The green and red points are the centroids of the guest molecules, and the light blue plane passes through a guest molecule. The dashed line color scheme is as follows: Hydrogen bonds between Q_8_H_8_ molecules: orange; twist angle: green; line vertically connecting a centroid to the molecular plane of another guest: blue. **a** Geometries of benzene dimers: (A) T-shaped, (B) parallel-displaced, (C) parallel-*D*_6h_, and (D) parallel-*D*_6d_. **b** polarized micrograph and **c** crystal photograph with cell axes, which were drawn by CrysAlisPro software; **d** 3D crystal packing; **e** hydrogen-bonding interactions; **f** 3D space-filling representation; **g** visualization of the solvent-accessible surface of the one-dimensional pores calculated using the software Olex2; **h** partial space-filling views of the stacked guest molecules; **i** top view and diagonal view of the geometry of the stacked guest molecules.

Numerous theoretical calculations of the intermolecular stabilizing interactions of benzene dimers have been carried out^25–33^ because interactions between π systems are important in the crystal packing of organic molecules that contain unsaturated moieties and in the three-dimensional structures of biological systems such as DNAs and proteins. Thus, detailed investigations of benzene dimers are important in order to understand π–π interactions. Based on previously reported theoretical calculations, the configurations of benzene dimers have been categorized into three main types: T-shaped, parallel-displaced, and parallel (Fig. 4a). These calculations revealed that the most favorable configurations are the T-shaped and parallel-displaced configurations. When comparing the stability of these three configurations, it becomes evident that the parallel configurations are most unfavorable on account of the electrostatic repulsion between the benzene molecules. T-shaped and parallel-displaced configurations have been experimentally observed in single crystals^34–36^. Parallel configurations can exhibit two types of geometries: parallel-*D*_6h_ and parallel-*D*_6d._ Their optimized intermolecular distance is approximately 3.7–3.9 Å^28^, and the optimized energy of parallel-*D*_6d_ is slightly more stable than that of parallel-*D*_6h_. These theoretical calculations, as well as the size and separation of the nano-honeycomb channel of the THF crystal discussed above, support the notion that the channel is appropriate for stacking benzene molecules in parallel.

Various experimental studies have investigated the confinement of benzene molecules in the nanospaces of zeolites^37^, MOFs^38^, hydrogen-bonded supramolecules^15^, and other structures. Moreover, Tachikawa et al. have theoretically investigated the ionization dynamics of a benzene cluster. They found that the benzene cluster cation could act as a molecular electronic device by changing the benzene configuration from T-shaped to parallel and that its ON–OFF switching behavior was accelerated by H_2_O molecules^39^.

A large amount of benzene was added to the THF solution of **1**, and the solvent was then slowly removed by evaporation at 5 °C to obtain columnar crystals (3D⊃benzene) in which benzene was confined and stacked in the nano-honeycomb pores (Fig. 4b–i). The stacked benzene molecules form infinite columns (Fig. 4d, h). Two co-crystallized molecules of benzene per molecule of **1** are confined in the nano-honeycomb-shaped pores in direction of the *c*-axis (Fig. 4c–e). The molecular plane of benzene is oriented orthogonally with respect to the long axis of the pore (Fig. 4c, d, f). The space-filling representation and surface visualization of the inclusion channels clearly show that the benzene molecules are tightly packed in the channels and positioned at the wide areas of the nano-honeycomb-shaped pores in an arrangement resembling bamboo joints (Fig. 4f–h). The channels have a star-shaped octagonal cross-section with a width ranging from 4.2 to 5.3 Å, and the ratio of solvent-accessible volume per unit cell is 43.5% (Fig. 4g). At the wide octagonal section, the longest diagonals are those connecting the four points at which the rod forms hydrogen bonds with neighboring rods within the network. The benzene molecule is packed so that four of the CHs are oriented along these two long diagonals to minimize steric hindrance. The closest distances between the oxygen atoms in **1** and atoms of the guest molecules in the 3D⊃guest crystals are summarized in Supplementary Table 14. In agreement with theoretical calculations and steric considerations, the parallel-*D*_6d_ geometry is observed in the 3D⊃benzene crystals (Fig. 4a(D)). The neighboring six-membered rings exhibit a dihedral angle of 29.3(3)° as determined from the angle between the planes passing through the four carbons in Fig. 4i. The distance between the centroids of the two adjacent benzene molecules is 3.7730(15) Å, i.e., equivalent to the vertical distance between the centroid of one benzene molecule and the molecular plane of a neighboring benzene molecule (Fig. 4i). The intermolecular distance between benzenes was highly consistent with the aforementioned theoretical calculations of parallel-*D*_6d_ (approx. 3.75–3.8 Å^26,27,30^). The distances and twist angle between two adjacent guest molecules in the 3D⊃guest crystals are summarized in Table 1. The single-crystal XRD analyses showed that the nano-honeycomb channel runs along the long axis of the columnar crystal (Fig. 4c). In the obtained 3D⊃benzene crystals, the length of the longest side ranged from 0.2 to 0.45 mm; thus, approximately 0.5–1.2 million benzene molecules are stacked in the channel, and each benzene stacking polymer is isolated by the framework. Although the main crystals are the target crystals, not only 3D⊃benzene crystals but also other crystals described in this paper were obtained as a mixture with different crystal morphologies. The separation of each crystal based on their crystal morphologies is virtually impossible given that the size of these crystals is too small to select. Moreover, when the crystals were left in the air, the guest molecules and co-crystallization solvents evaporated immediately. Therefore, accurate yields cannot be calculated.

**Table 1:** Distances and twist angle between two adjacent guest molecules in 3D⊃guest crystals.

Crystal	Centroid–centroid (Å)	Vertical distances (Å)	Twist angle (°)
3D⊃benzene	3.7730 (15)	3.7730 (15)	29.3 (3)
3D⊃thiophene	3.7743 (14)–3.821(7)	3.77265 (10)	
3D⊃selenophene	3.779 (7)–3.788(10)	3.7705 (3)	
3D⊃*p*-benzoquinone	3.7561 (9)	3.7561 (9)	33.7 (2)
3D⊃THF·*p*-benzoquinone	3.4882 (3)	3.48397 (14)^a^	
3D⊃thiophene·*p*-benzoquinone	3.7711 (18)	3.7680 (15)	
3D⊃benzene·*p*-benzoquinone	3.7636 (5)	3.7636 (5)	

^a^Distance between the centroid of THF and the *p*-benzoquinone plane.

### Spectroscopic analysis of 3D⊃benzene crystals

**Fig. 5:**
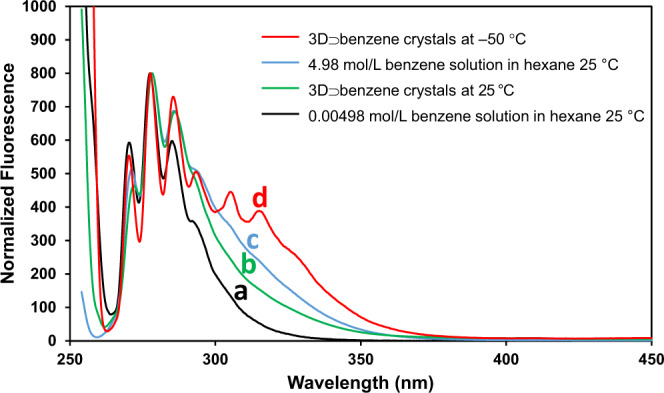
Fluorescence spectra at room temperature (*λ*_excitation_ = 254 nm), normalized using the peak at 278.6 nm. The background corrections of all the emission and excitation spectra were performed using a 5.5 g/L solution of rhodamine B in ethylene glycol (200–400 nm) and a halogen lamp (JASCO ESC-842, 20 W, 400–850 nm). A 0.00498 mol/L benzene solution in hexane at 25 °C (a), 3D⊃benzene crystals at 25 °C (b), 4.98 mol/L benzene solution in hexane at 25 °C (c), 3D⊃benzene crystals at −50 °C (d).

When the crystals were left in the air, the benzene molecules evaporated immediately. Thus, we coated a crystal with 1,3-dimethyladamantane and measured its ultraviolet-visible (UV–Vis) and fluorescence spectra. In the solid-state UV–Vis spectrum of 3D⊃benzene, a broad absorption centered at 255.5 nm, as well as the absorption peaks typical for benzene, were observed (Supplementary Fig. 3). On the other hand, not only the monomer fluorescence but also the excimer fluorescence of benzene was observed for the crystal of 3D⊃benzene at room temperature (Fig. 5). As shown in Fig. 5, although the strength of the excimer emission from the 3D⊃benzene crystals is weaker than that of a hexane solution of benzene with the same concentration (4.98 mol/L), the existence of excimer fluorescence from the 3D⊃benzene crystals at approximately 325 nm can be confirmed unambiguously at 25 °C, while in a hexane solution with a concentration of 0.00498 mol/L, no excimer emission was observed^40^. The excimer fluorescence could be clearly observed at −50 °C (Fig. 5)^41^. The excimer fluorescence of benzene is a relaxation emission from an excited dimeric benzene species composed of excited-state benzene and ground-state benzene stacked in a parallel face-to-face fashion with a distance close to 3.05 Å^42^. The excimer fluorescence provides evidence that the benzene molecules are not tightly fixed at the wide areas of the nano-honeycomb pores but could approach each other sufficiently close to form π–π interactions. The single-crystal XRD analysis results, spectroscopic observations, and many previously reported theoretical calculations strongly support the conclusion that parallelly and infinitely stacked benzene molecules in 3D⊃benzene mutually interact through their π systems in a nano-honeycomb pore.

Crystals of 3D⊃benzene were subjected to thermogravimetry-differential thermal analysis (TG-DTA) measurements to examine their stability. The obtained results suggest that benzene molecules evaporate below 102 °C and that the dehydrative condensation of the silanol framework occurs over 141 °C (Supplementary Fig. 4). Furthermore, a powder XRD analysis was carried out on a crystalline sample of 3D⊃benzene from which benzene had been removed by exposure to reduced pressure (400 Pa) for 27 h at room temperature. The powder XRD spectrum showed prominent diffraction peaks and the signal pattern was similar to the simulated pattern based on the data obtained from the single-crystal XRD analysis of 3D⊃benzene (Supplementary Fig. 5).

### Preparation and structural analysis of three-dimensional 3D⊃thiophene, 3D⊃selenophene, 3D⊃*p*-benzoquinone, 3D⊃THF·*p*-benzoquinone, and 3D⊃thiophene·*p*-benzoquinone crystals

**Fig. 6:**
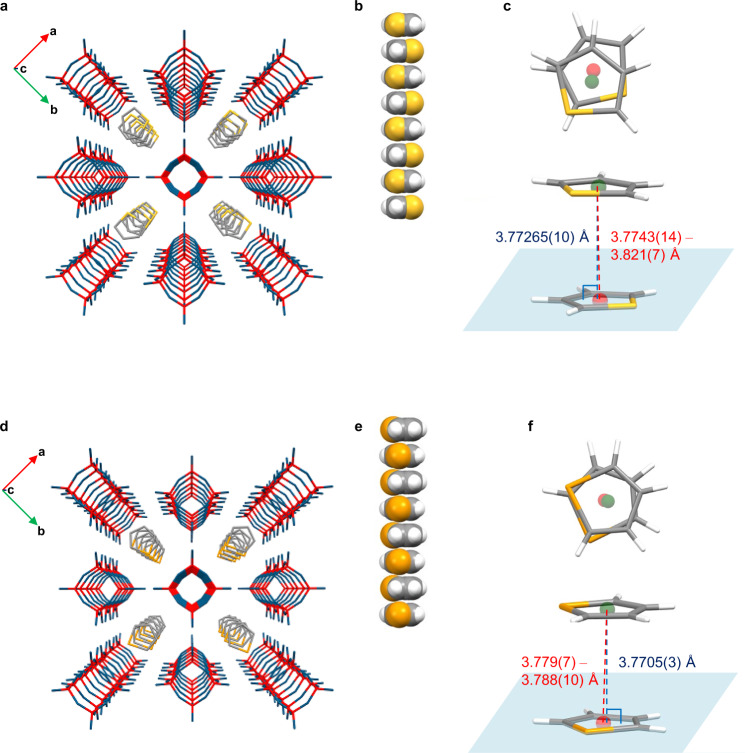
Crystal structures of 3D crystals encapsulating heteroaromatic molecules. Crystal structures of 3D⊃thiophene (**a**–**c**) and 3D⊃selenophene (**d**–**f**) determined by single-crystal X-ray diffraction. The orientation of the guest molecules in 3D⊃thiophene and 3D⊃selenophene was disordered, and a representative orientation is shown for clarity. The hydrogen atoms in the 3D crystal packing representations have been omitted for clarity. Color code: Si = red; O = blue; C = gray; H = white; S = yellow; Se = orange. The green and red points are the centroids of the guest molecules, and the light blue plane passes through a guest molecule. The dashed line color scheme is as follows: a line connecting the centroids of two guests: red; line vertically connecting a centroid to the molecular plane of another guest: blue. **a**, **d** 3D crystal packing; **b**, **e** partial space-filling views of the stacked guest molecules; **c**, **f** top view and diagonal view of the geometry of the stacked guest molecules.

Next, we turned our attention to encapsulating heteroaromatic molecules in the nano-honeycomb pores and investigated thiophene and selenophene, for which the distance between the most distant hydrogens in the molecule was calculated to be 4.57 and 4.69 Å, respectively. In the same manner as the 3D⊃benzene crystals, **1**·2thiophene or [Si_8_O_12_][OH]_8_·2thiophene (3D⊃thiophene) and **1**·2selenophene or [Si_8_O_12_][OH]_8_·2selenophene (3D⊃selenophene) crystals with thiophene and selenophene molecules encapsulated in the nano-honeycomb pores, respectively, were obtained by recrystallization (Fig. 6). The thiophene and selenophene molecules are rotationally disordered, with four equivalent components in which the bulky S and Se atoms are oriented toward the four largest vertices of the wide section and the S and Se atoms coordinate to Si–OH to obtain a certain extent of stabilization. The thiophene dimer has been the subject of theoretical calculations, and the results suggested that the T-shaped configuration is more favorable than the parallel-displaced configuration due to the considerable stabilization via electrostatic and dispersion interactions^43^. In thiophene crystals, the T-shaped intermolecular configuration has been experimentally observed at low temperatures and ordinary pressures^44^. In 3D⊃thiophene and 3D⊃selenophene, the thiophene and selenophene molecules are stacked in parallel (Fig. 6a, b, d, e), but their centroids do not eclipse along the *c*-axis (Fig. 6c, f). The distance between the centroids of two adjacent thiophene and selenophene molecules are 3.7743(14)–3.821(7) and 3.779(7)–3.788(10) Å, respectively, and the respective vertical distances from one centroid to the molecular plane of another guest are 3.77265(10) and 3.7705(3) Å (Fig. 6c, f and Table 1).

**Fig. 7:**
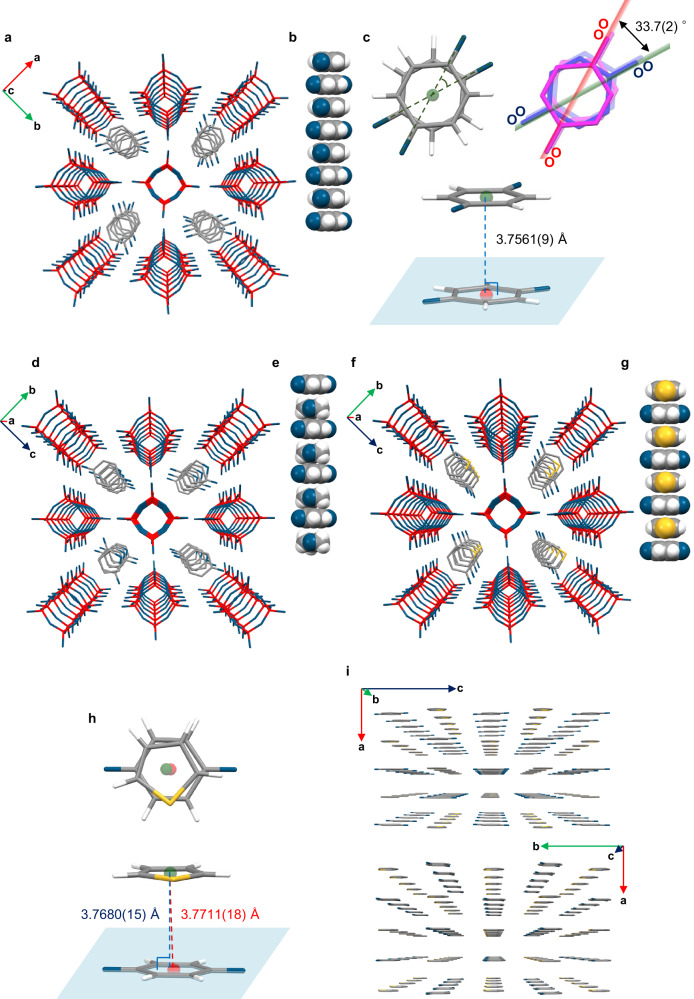
Crystal structures of 3D crystals encapsulating *p*-benzoquinone. Crystal structures of 3D⊃*p*-benzoquinone (**a**–**c**), 3D⊃THF·*p*-benzoquinone (**d**, **e**), and 3D⊃thiophene·*p*-benzoquinone (**f**–**i**) determined by single-crystal X-ray diffraction. The orientation of the guest molecules in 3D⊃THF·*p*-benzoquinone and 3D⊃thiophene·*p*-benzoquinone was disordered, and a representative orientation is shown for clarity. The hydrogen atoms in the 3D crystal packing representations have been omitted for clarity. Color code: Si = red; O = blue; C = gray; H = white; S = yellow. The green and red points are the centroids of the guest molecules, and the light blue plane passes through a guest molecule. The dashed line color scheme is as follows: twist angle: green; line connecting the centroids of two guests: red; line vertically connecting a centroid to the molecular plane of another guest: blue. **a**, **d**, **f** 3D crystal packing; **b**, **e**, **g** partial space-filling views of the stacked guest molecules; **c**, **h** top view and diagonal view of the geometry of the stacked guest molecules; **i** 3D crystal packing of guest molecules in the crystal of 3D⊃thiophene·*p*-benzoquinone with **1** omitted for clarity.

As shown in Fig. 7a–c, when *p*-benzoquinone, which is not an aromatic compound but instead an unsaturated cyclic molecule, was incorporated as the guest molecule, the *p*-benzoquinone molecules also stacked parallelly in the elliptical nano-honeycomb pore (**1**·2*p*-benzoquinone or [Si_8_O_12_][OH]_8_·2*p*-benzoquinone (3D⊃*p*-benzoquinone)) in contrast to single crystals of *p*-benzoquinone with an expanded C–H···O hydrogen-bond network in the in-plane direction^45,46^. The calculated oxygen–oxygen distance in a *p*-benzoquinone molecule is 5.34 Å, making it a larger molecule than benzene. Yellow single crystals of 3D⊃*p*-benzoquinone were obtained by evaporating a THF solution of **1** and *p*-benzoquinone at 5 °C. X-ray crystal analysis of a 3D⊃*p*-benzoquinone crystal clearly indicated that the nano-honeycomb pore is flexible and that its shape can change depending on the guest molecules by rotating around the center axis of the rod. The distance between the centroids of the six-membered rings of neighboring *p*-benzoquinone molecules is 3.7561(9) Å, and the dihedral angle is 33.7(2)° (Table 1), as determined from the angles between the planes passing through the four oxygens of the benzoquinones in Fig. 7c.

Unexpectedly, **1**·THF·*p*-benzoquinone (3D⊃THF·*p*-benzoquinone) crystals in which *p*-benzoquinone and THF molecules alternate along the direction of the *a*-axis were obtained simultaneously using the same recrystallization conditions (Fig. 7d, e and Table 1).

Inspired by this result, we reasoned that if a planar π molecule of the same size as THF was used, it might exhibit alternating π-stacking with *p*-benzoquinone. We then used thiophene instead of THF. As expected, **1**·thiophene·*p*-benzoquinone or [Si_8_O_12_][OH]_8_·thiophene·*p*-benzoquinone (3D⊃thiophene·*p*-benzoquinone) crystals in which *p*-benzoquinone and thiophene molecules are alternately stacked in the pores were also obtained by evaporating a THF solution of **1** including *p*-benzoquinone and thiophene (Fig. 7f–i). In the direction of the *a*-axis, an alternate arrangement of thiophene or THF molecules and *p*-benzoquinone molecules was observed (Fig. 7e, g). On the other hand, focusing on the surface formed by the *b*-axis and the *c*-axis and ignoring the framework, lines of *p*-benzoquinone and lines of thiophene or THF are alternately arranged in direction of the *b*-axis or *c*-axis (Fig. 7i and Supplementary Fig. 6). The distance between the centroids of neighboring thiophene and *p*-benzoquinone molecules is 3.7711(18) Å, and the vertical distance from one centroid to the molecular plane of another guest is 3.7680(15) Å (Fig. 7h and Table 1). Based on their intermolecular distance and arrangement, there appears to be some π–π interaction between *p*-benzoquinone and thiophene. The coordination ability of *p*-benzoquinone is not sufficiently strong to break the SiO–H hydrogen-bonding networks; however, its affinity for SiO–H is higher than that of thiophene, and therefore, *p*-benzoquinone can be encapsulated in the framework even when present in a very small molar ratio relative to thiophene in solution. The alternating arrangement of thiophene or THF and *p*-benzoquinone was attributed to the different molecular sizes of thiophene, THF, and *p*-benzoquinone. During the growth of the crystal, solvent molecules with a size suitable for the shape of the pore are selectively arranged one after another in a molecular-recognition-like manner, and as a result, an alternately arranged structure is obtained.

In the same manner, as the 3D⊃thiophene·*p*-benzoquinone crystals, **1**·benzene·*p*-benzoquinone or [Si_8_O_12_][OH]_8_·benzene·*p*-benzoquinone (3D⊃benzene·*p*-benzoquinone) crystals, which encapsulate *p*-benzoquinone and benzene, were obtained from recrystallizing a THF solution of **1** that contained *p*-benzoquinone and benzene (Supplementary Fig. 7 and Table 1). From the electron density determined by the XRD analysis, the ratio of encapsulated benzene to *p*-benzoquinone molecules was estimated to be roughly 67:33. Although Manojkumar et al. and Tsuzuki et al. have carried out theoretical calculations of benzene-*p*-benzoquinone clusters and reported that the parallel-displaced geometry was the most preferred structure^47,48^ in these crystals, the benzene and *p*-benzoquinone molecules seem to be parallel-stacked to some extent.

In summary, we found that cubic octamer of orthosilicic acid **1** precisely assembles in crystals to form HIFs with various types of hydrogen-bonding networks. We will investigate the physical properties of these parallel-stacked molecules in detail in the near future. We expect that the results will lead to a wide variety of high-performance and functional silica-based materials and advanced organic–inorganic hybrid materials with well-defined structures. Such materials can also be expected to contribute to the elucidation of novel properties of common small molecules stacked in parallel and open new fields in molecular electronic devices.

## Methods

### General information

^1^H (600 MHz), ^13^C (151 MHz), and ^29^Si (119 MHz) NMR spectra were measured in methanol-*d*_4_, acetone-*d*_6_, THF-*d*_*8*_, and *N*,*N*-dimethylformamide-*d*_7_ using a Bruker AVANCE III spectrometer equipped with a CryoProbe. Chemical shifts (*δ*) are reported in parts per million (p.p.m.) relative to an internal standard (trimethylphenylsilane, 0.26 p.p.m. for ^1^H and −4.2 p.p.m. for ^29^Si for 0D; 1,4-bis(trimethylsilyl)benzene, 0.25 p.p.m. for ^1^H and −4.3 p.p.m. for ^29^Si for 3D⊃THF).

UV–Vis diffuse transmittance spectroscopy was performed on a JASCO V-750 instrument equipped with a horizontal integrating sphere (JASCO PIV-756). Fluorescence spectra were measured using a JASCO FP-8500 with an FLH-809 film holder accessory. An Oxford instruments Optistat DN was used for temperature-dependent measurements. The background correction of all the emission and excitation spectra was carried out using a combination of a 5.5 g/L rhodamine B ethylene glycol solution (200–400 nm) and a halogen lamp (JASCO ESC-842, 20 W, 400–850 nm). GPC was performed using a Japan Analytical Industry LC5060 instrument with DMAc as the eluent. Polarized micrographs of the crystals were obtained using an Olympus SZX10 microscope equipped with a DP74-CU color camera. TG-DTA measurements were conducted on a Hitachi High-Tech Science STA7200 RV instrument in dry air from 30 to 600 °C at a flow rate of 200 mL/min and a heating rate of 2.5 °C/min. XRD measurements were conducted on a Rigaku R-AXIS IV X-ray diffractometer using CuKα radiation (*λ* = 0.1542 nm, 40 kV, 30 mA) monochromated using graded elliptic multilayer optics. The sample was transferred into the bottom of a quartz capillary (glass; outer diameter: 1.5 mm; wall thickness: 0.01 mm) and sealed in the capillary under a dry N_2_ gas atmosphere. Diffraction patterns were recorded using an imaging plate (Fuji-Photo Film, 300 mm × 300 mm) in a flat camera (camera length: 150.0 mm). The measurements were carried out under a 300 s exposure. The 2D diffraction images were converted to 2*θ* − *I* plots.

TMU (>98.0%), trimethylphenylsilane (>98%), Meldrum’s acid (>98.0%), thiophene (>98.0%), selenophene (>98.0%), 1,3-dimethyladamantane (>99.0%), and tetramethylammonium hydroxide (ca. 25% in water) were obtained from Tokyo Chemical Industry Co., Ltd. Nitric acid (68.6–70.4%), sulfuric acid (95+%), aniline, DMAc (99.5+%), THF (dehydrated, stabilizer free, 99.5+%), Et_2_O (99.5+%), diglyme (98.0+%), acetonitrile (99.5+%), *p*-benzoquinone (98+%), benzene (for the synthesis of 3D⊃benzene, 99.5+%), benzene (for the spectrochemical analysis, 99.8+%), hexane (for the spectrochemical analysis, 96.0+%), *N*,*N*-dimethylformamide-*d*_7_ (99.5+%), methanol-*d*_4_ (99.8%), THF-*d*_8_ (99.5%), and silica gel (Wakogel 60N) were obtained from FUJIFILM Wako Pure Chemical Co. Acetone-*d*_6_ (99.9%) was obtained from Kanto Chemical Co., Inc. Parabar 10312 was obtained from Hampton Research Inc. DFT calculations were carried out using the Gaussian 09 program package^24^.

In the bulk [Si_8_O_12_][OH]_8_·*n*DMAc crystals, the number of DMAc molecules per molecule of **1** (*n* in **1**·*n*DMAc) varied (*n* = 5–14) depending on the purification conditions, e.g., the time taken to evaporate the solvent. The number of DMAc molecules contained in the bulk crystals was determined by integrating the signal of the three protons of the acetyl group of DMAc in the ^1^H NMR spectrum.

Although the main crystals are the target crystals, all crystals described in this paper were obtained as a mixture with different crystal morphologies. Furthermore, the separation of each crystal based on their crystal morphologies is virtually impossible given that the size of these crystals is too small to select. Moreover, when the crystals were left in the air, the guest molecules and co-crystallization solvents evaporated immediately. Therefore, the accurate yields of all crystals cannot be calculated.

### Preparation of **2**

**2** was prepared according to a literature procedure by the reaction of silica gel and tetramethylammonium hydroxide in water^49^. A mixture of silica gel (Wakogel 60N: 304.72 g, 5.07 mol) and an aqueous solution of 20% tetramethylammonium hydroxide (2080 g, 4.56 mol as N(CH_3_)_4_OH) was stirred at 25 °C. The solution was stirred for 6 h and then kept at 4 °C in a refrigerator for 2 days. Crystalline precipitate was filtered and washed with a small amount of acetone. After drying in vacuo, colorless crystals of [Si_8_O_20_][NMe_4_]_8_·53.6H_2_O (833.74 g, 69.5% yield) were obtained as a first crop. Second crop crystals, [Si_8_O_20_][NMe_4_]_8_·58.4H_2_O (249.04 g, 20.0% yield), were also obtained from the further recrystallization. The total reaction yield was 89.5%. The formula weight, i.e., the number of water molecules, of these crystals were determined by titration using 0.1 mol/L aqueous HNO_3_ with phenolphthalein as an indicator. In the bulk **2** crystals, the number of water molecules (*n* = ca. 60) varied depending on the drying process and time. The number of water molecules contained in the bulk crystals was determined based on the elemental analysis.

[Si_8_O_20_][NMe_4_]_8_. ^1^H NMR (methanol-*d*_4_): δ 5.45 (N(C*H*_3_)_4_, 96H); ^13^C NMR (methanol-*d*_4_): 56.7 (N(*C*H_3_)_3_); ^29^Si NMR (methanol-*d*_4_): −99.0.

### Preparation of 0D using Meldrum’s acid

0D was prepared according to a published procedure by the ammonium–proton exchange of the ammonium silicate **2** with Meldrum’s acid^22^. Meldrum’s acid (363 mg, 2.52 mmol) was added to a suspension of [Si_8_O_20_][NMe_4_]_8_·55.4H_2_O (640 mg, 0.300 mmol) in DMAc (8 mL) in a vial equipped with a magnetic stirring bar. After stirring at 25 ˚C for 10 min, the mixture was purified by preparative GPC and recrystallization, which afforded colorless crystals of **1**·9.0DMAc (based on the ^1^H NMR spectra; 390 mg, 93% yield).

[Si_8_O_12_][OH]_8_. ^1^H NMR (THF-*d*_*8*_): 6.41 (SiO*H*); ^29^Si NMR (THF-*d*_*8*_): −100.0 (*Si*OH); HRMS (ESI) *m*/*z* calcd. for Si_8_O_20_H_7_ 550.7690 [M − H]^−^, found 550.7694.

### Representative procedure for the preparation of 1 using nitric acid as an acid in THF and crystallization of 0D

Nitric acid (8.92 mL, 141.6 mmol) was added to a suspension of [Si_8_O_20_][NMe_4_]_8_·24.3H_2_O (25.21 g, 16.0 mmol) in THF (600 mL) in a conical flask equipped with a magnetic stirring bar. After stirring at room temperature for 30 min, the mixture was filtered by suction filtration. DMAc (160 mL) was added to the filtrate, and the residue was distilled under reduced pressure to exchange the solvent. Recrystallization from Et_2_O (poor solvent method) in a DMAc solution yielded colorless crystals of 0D (based on the ^1^H and ^29^Si NMR spectra; 18.42 g, 82% yield).

[Si_8_O_12_][OH]_8_. ^1^H NMR (acetone-*d*_*6*_): δ 6.78 (SiO*H*, 8H); ^29^Si NMR (acetone-*d*_*6*_): −100.2 (*Si*OH) (Supplementary Fig. 1).

### Preparation of single crystals of 1D-C

0D (112 mg) was dissolved in diglyme (0.8 mL) at 45 °C and allowed to stand at 4 °C to obtain single crystals of 1D-C.

### Preparation of single crystals of 1D-W

0D (112 mg) was dissolved in THF (2.0 mL) and allowed to stand at −30 °C to obtain single crystals of 1D-W.

### Preparation of single crystals of 1D-R

Nitric acid (3.04 mL, 48.229 mmol) was added to a suspension of [Si_8_O_12_][ONMe_4_]_8_·48.7H_2_O (8.070 g, 4.005 mmol) in THF (150 mL) in a conical flask equipped with a magnetic stirring bar. After 30 min of stirring at room temperature, the mixture was filtered by suction filtration. TMU (20.0 mL) was added to the filtrate and the residue was distilled under reduced pressure to exchange the solvent. Colorless crystals of 1D-R for XRD analysis were obtained from the TMU solution by the vapor-diffusion method using Et_2_O as a poor solvent.

### Preparation of single crystals of 2D-S

Nitric acid (3.04 mL, 48.229 mmol) was added to a suspension of [Si_8_O_12_][ONMe_4_]_8_·48.7H_2_O (8.069 g, 4.004 mmol) in THF (150 mL) in a conical flask equipped with a magnetic stirring bar. After 30 min of stirring at room temperature, the mixture was filtered by suction filtration. Diglyme (30.0 mL) was added to the filtrate and the residue was distilled under reduced pressure to exchange the solvent. Colorless crystals of 2D-S were obtained from the diglyme solution by the vapor-diffusion method using Et_2_O as a poor solvent. The colorless crystals were washed with Et_2_O and then stored in Et_2_O.

### Preparation of single crystals of 2D-MS

0D (112 mg) was dissolved in diglyme (1.2 mL) at 45 °C and allowed to stand at −18 °C to obtain colorless crystals of 1D-W.

### Preparation of single crystals of 3D⊃THF

Nitric acid (0.239 mL, 3.792 mmol) was added to a suspension of [Si_8_O_12_][ONMe_4_]_8_·54.0H_2_O (0.847 g, 0.401 mmol) in THF (20.0 mL) in a conical flask equipped with a magnetic stirring bar. After 30 min of stirring at room temperature, the mixture was filtered by suction filtration. The filtrate was recrystallized by evaporating the solvent at 30 °C to obtain colorless crystals of 3D⊃THF. The crystals were washed with THF and then stored in THF (Supplementary Fig. 2a–g).

### Preparation of single crystals of 3D⊃benzene

Nitric acid (3.05 mL, 48.388 mmol) was added to a suspension of [Si_8_O_12_][ONMe_4_]_8_·48.7H_2_O (8.102 g, 4.021 mmol) in THF (150 mL) in a conical flask equipped with a magnetic stirring bar. After stirring at room temperature for 30 min, the mixture was filtered by suction filtration to give 155.30 g of a THF solution of **1** as the filtrate. To the 19.68 g of filtrate, benzene (5.0 mL) was added. Colorless crystals of [Si_8_O_12_][OH]_8_·2benzene, 3D⊃benzene, were obtained by evaporating the solvent at 5 °C. The colorless crystals were washed with acetonitrile and then stored in benzene. The solid-state UV–Vis spectrum of 3D⊃benzene is shown in Supplementary Fig. 3.

### Preparation of single crystals of 3D⊃thiophene

Nitric acid (3.05 mL, 48.377 mmol) was added to a suspension of [Si_8_O_12_][ONMe_4_]_8_·48.7H_2_O (8.119 g, 4.029 mmol) in THF (150 mL) in a conical flask equipped with a magnetic stirring bar. After stirring at room temperature for 30 min, the mixture was filtered by suction filtration to give 153.04 g of a THF solution of **1** as the filtrate. To the 19.11 g of filtrate, thiophene (2.0 mL) was added. Colorless crystals of [Si_8_O_12_][OH]_8_·2thiophene, 3D⊃thiophene, were obtained by evaporating the solvent at 5 °C. The crystals were washed with acetonitrile and then stored in thiophene.

### Preparation of single crystals of 3D⊃selenophene

Nitric acid (0.401 mL, 6.368 mmol) was added to a suspension of [Si_8_O_12_][ONMe_4_]_8_·24.3H_2_O (0.793 g, 0.503 mmol) in THF (18.8 mL) in a conical flask equipped with a magnetic stirring bar. After 30 min of stirring at room temperature, magnesium sulfate (1.21 g) was added, and after another 10 min of stirring, the mixture was filtered by suction filtration. To the filtrate, selenophene (1.5 mL) was added. Yellow crystals of 3D⊃selenophene were obtained by evaporating the solvent at 15 °C. The crystals were washed with acetonitrile and then stored in selenophene.

### Preparation of single crystals of 3D⊃*p*-benzoquinone with 3D⊃THF·*p*-benzoquinone

Nitric acid (3.04 mL, 48.229 mmol) was added to a suspension of [Si_8_O_12_][ONMe_4_]_8_·54.0H_2_O (8.069 g, 3.823 mmol) in THF (150 mL) in a conical flask equipped with a magnetic stirring bar. After 30 min of stirring at room temperature, the mixture was filtered by suction filtration to give 150.82 g of a THF solution of **1** as the filtrate. To the 9.32 g of filtrate, *p*-benzoquinone (109.1 mg, 0.99 mmol) was added. Yellow crystals of 3D⊃*p*-benzoquinone with 3D⊃THF·*p*-benzoquinone were obtained by evaporating the solvent at 5 °C. The crystals were washed with acetonitrile, dried, and stored.

### Preparation of single crystals of 3D⊃thiophene·*p*-benzoquinone with 3D⊃THF·*p*-benzoquinone

Nitric acid (0.775 mL, 12.290 mmol) was added to a suspension of [Si_8_O_12_][ONMe_4_]_8_·48.7H_2_O (2.047 g, 1.016 mmol) in THF (37.5 mL) in a conical flask equipped with a magnetic stirring bar. After 30 min of stirring at room temperature, the mixture was filtered by suction filtration. To the filtrate, *p*-benzoquinone (109.0 mg, 0.99 mmol) and thiophene (10.0 mL) were added. The bright yellow crystals of 3D⊃thiophene·*p*-benzoquinone with 3D⊃THF·*p*-benzoquinone were obtained by evaporating the solvent at 5 °C. The crystals were washed with acetonitrile, dried, and stored.

### Preparation of single crystals of 3D⊃benzene·*p*-benzoquinone with 3D⊃THF·*p*-benzoquinone

Nitric acid (3.08 mL, 48.935 mmol) was added to a suspension of [Si_8_O_12_][ONMe_4_]_8_·48.7H_2_O (8.146 g, 4.043 mmol) in THF (150 mL) in a conical flask equipped with a magnetic stirring bar. After stirring at room temperature for 30 min, the mixture was filtered by suction filtration. To the filtrate, *p*-benzoquinone (55.0 mg, 0.50 mmol) and benzene (5.0 mL) were added. The bright yellow crystals of 3D⊃benzene·*p*-benzoquinone with 3D⊃THF·*p*-benzoquinone were obtained by evaporating the solvent at 5 °C. The crystals were washed with acetonitrile, dried, and stored.

## Supplementary information

Supplementary Information

## Data Availability

X-ray crystallographic data are available free of charge from the Cambridge Crystallographic Data Centre under the reference numbers CCDC 2074152–2074164 (see Supplementary Table 15) via https://www.ccdc.cam.ac.uk/structures/.
