# Supplementary material for: Parallel-stacked aromatic molecules in hydrogen-bonded inorganic frameworks
Source: Nat Commun. 2021 Dec 10;12:7025. doi: 10.1038/s41467-021-27324-2 (PMC8664825; doi:10.1038/s41467-021-27324-2)
Supplement: Supplementary file 1 — Supplementary Information [file 41467_2021_27324_MOESM1_ESM.pdf]

Supplementary Information

Parallel-stacked aromatic molecules in hydrogen-bonded inorganic frameworks

Masayasu Igarashi*[1], Takeshi Nozawa[1], Tomohiro Matsumoto[1], Fujio Yagihashi[1], Takashi Kikuchi[2], Kazuhiko Sato*[1]

[1]Interdisciplinary Research Center for Catalytic Chemistry, National Institute of Advanced Industrial Science and Technology (AIST), Tsukuba Central 5, 1-1-1 Higashi, Tsukuba, 305-8565, Japan.
[2]Rigaku Corporation, 3-9-12 Matsubara-cho, Akishima-shi, Tokyo, 196-8666, Japan.

Corresponding author: masayasu-igarashi@aist.go.jp (M.I.); k.sato@aist.go.jp (K.S.)

Table of Contents

1. NMR, UV-Vis, TG-DTA and Powder XRD spectra 2–7
2. X-ray crystallography and data 8–25
3. Supplementary References 26

1. NMR and UV-Vis spectra

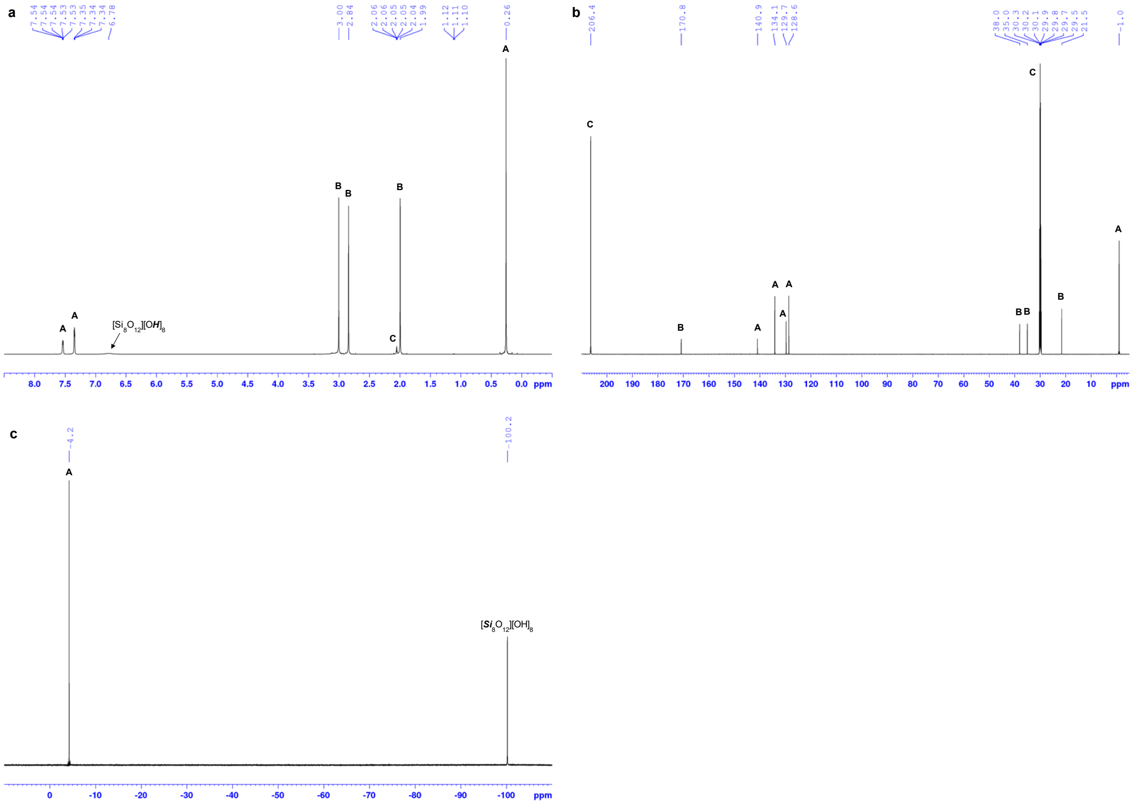

Supplementary Figure 1 | NMR spectra of [Si_8O_{12}][OH]$_8$·10DMAc crystals synthesized using nitric acid in THF. a) ^{1}H NMR spectrum (acetone-d_6, 600 MHz, 25 °C). The signal of **1** can be clearly observed. Other signals: **A:** standard ((CH_3)$_3$SiC_6H_5); **B:** co-crystallization solvents (DMAc); **C:** solvent (acetone). **b** ^{13}C NMR spectrum (acetone-d_6, 151 MHz, 25 °C). Signals: **A:** standard ((CH_3)$_3$SiC_6H_5); **B:** co-crystallization solvents (DMAc); **C:** solvent (acetone). **c** ^{29}Si NMR spectrum (acetone-d_6, 119 MHz, 25 °C). **A:** standard ((CH_3)$_3$SiC_6H_5).

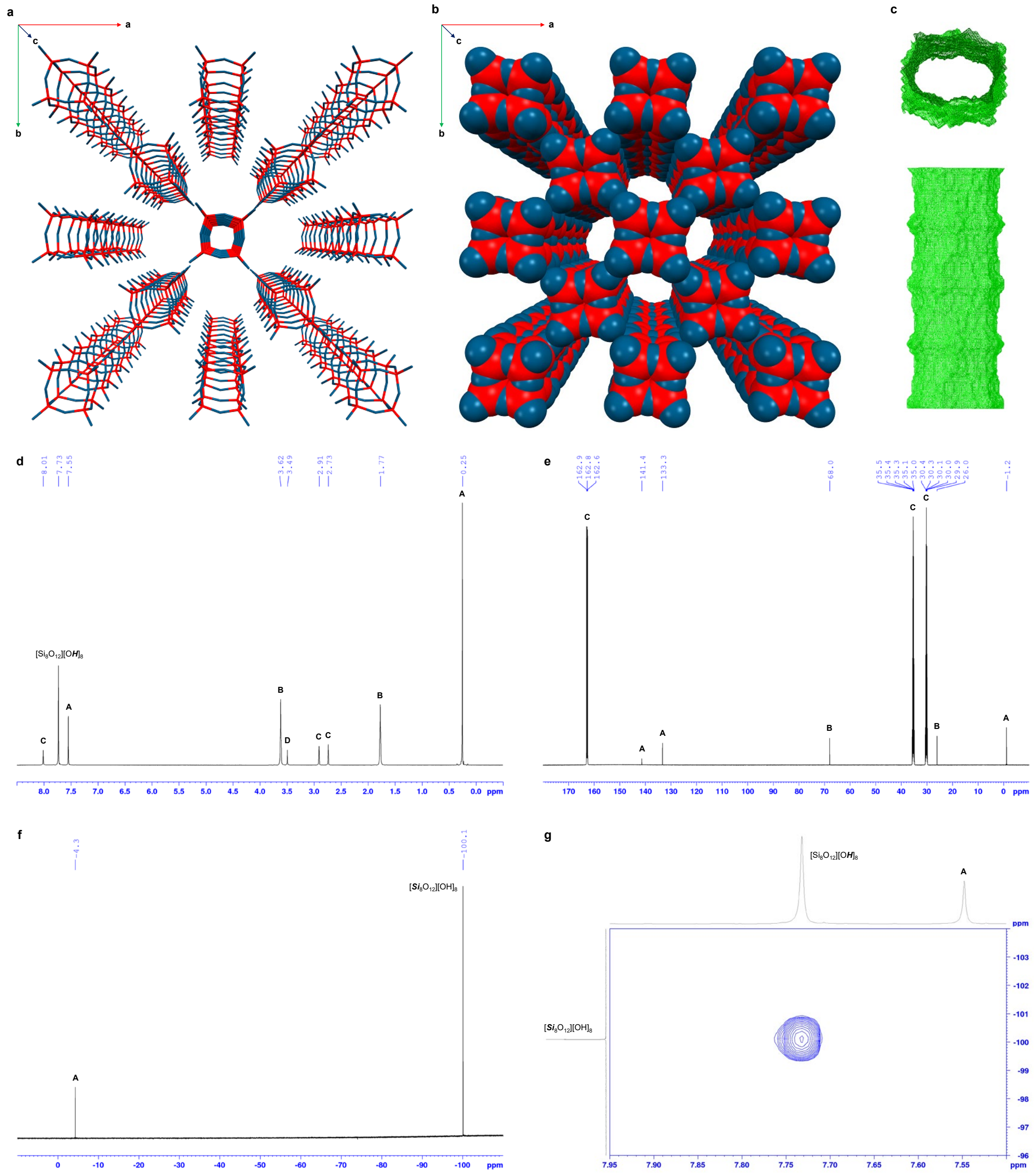

Supplementary Figure 2 | Crystal structure of 3D⊃THF determined by single-crystal X-ray diffraction and its NMR spectra after re-dissolution in DMF-d_6. Color code for **a**) and **b**): Si = red, O = blue; hydrogen atoms of the framework and THF molecules have been omitted for clarity. Crystal structure of 3D⊃THF showing **a**) 3D crystal packing (capped-sticks model); **b**) 3D crystal packing (space-filling model); **c**) visualization of the surface of one-dimensional pores calculated using Olex2. **d**) ^{1}H NMR spectrum (DMF-d_6, 600 MHz, 25 °C). The signal for **1** can be clearly observed. Other signals: **A:** standard $(1,4\text{-}\{(CH_3)_3Si\}_2C_6H_4)$; **B:** THF (guest molecule confined to

the nano-honeycomb channels); **C:** solvent (DMF); **D:** residual water molecules in the deuterated solvents. **e**) ^{13}C NMR spectrum (DMF-d_6, 151 MHz, 25 °C). Signals: **A:** standard (1,4-$\{(CH_3)_3Si\}_2C_6H_4$); **B:** THF; **C:** solvent (DMF). **f)** ^{29}Si NMR spectrum (DMF-d_6, 119 MHz, 25 °C). The signal for **1** can be clearly observed. **A:** standard (1,4-$\{(CH_3)_3Si\}_2C_6H_4$); **g)** ^{1}H–^{29}Si heteronuclear multiple-bond correlation (HMBC) NMR spectrum (DMF-d_6, 600 MHz, 25 °C). **A:** standard (1,4-$\{(CH_3)_3Si\}_2C_6H_4$). A cross peak is clearly observed between the ^{1}H and ^{29}Si signals of **1**.

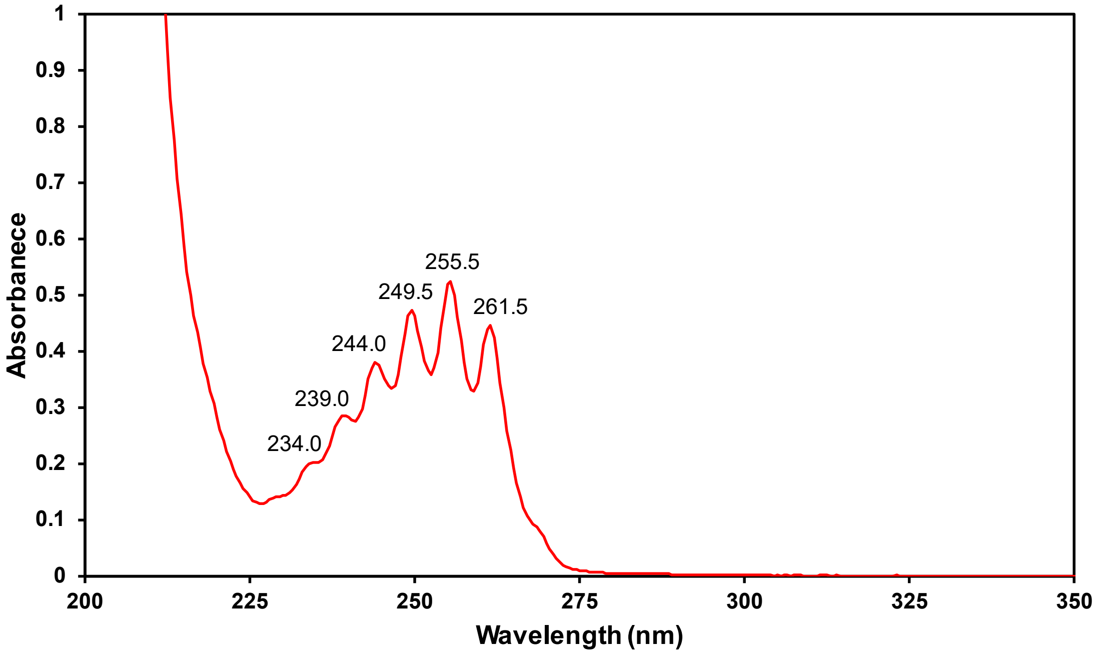

Supplementary Figure 3 | Solid state UV-Vis spectrum of a 3D⊃benzene crystal.

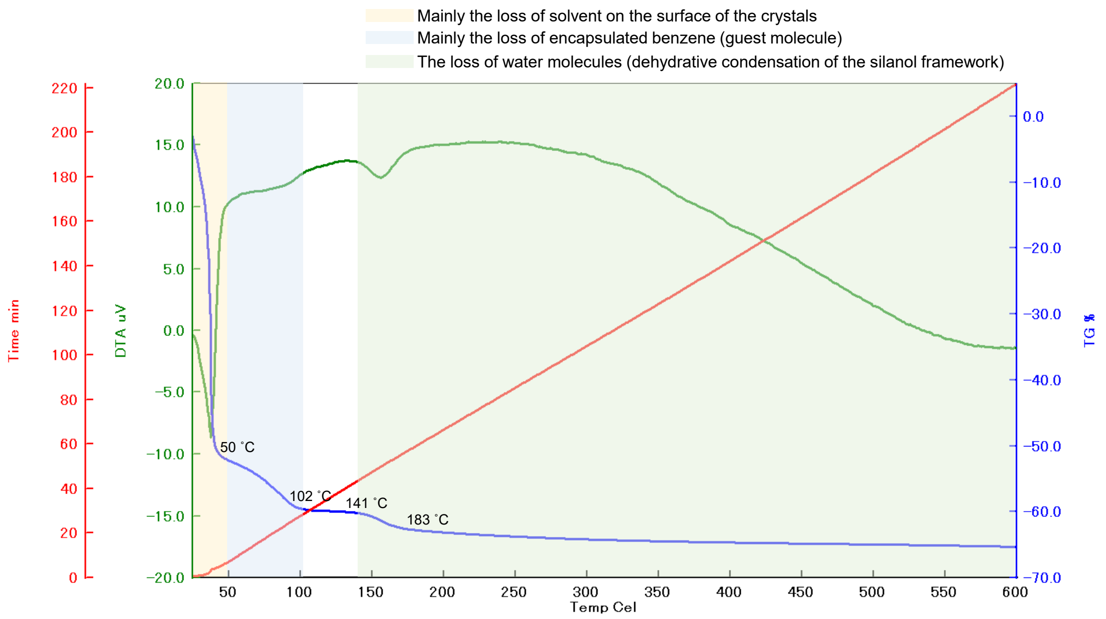

Supplementary Figure 4 | TG-DTA spectrum of a 3D⊃benzene crystals.

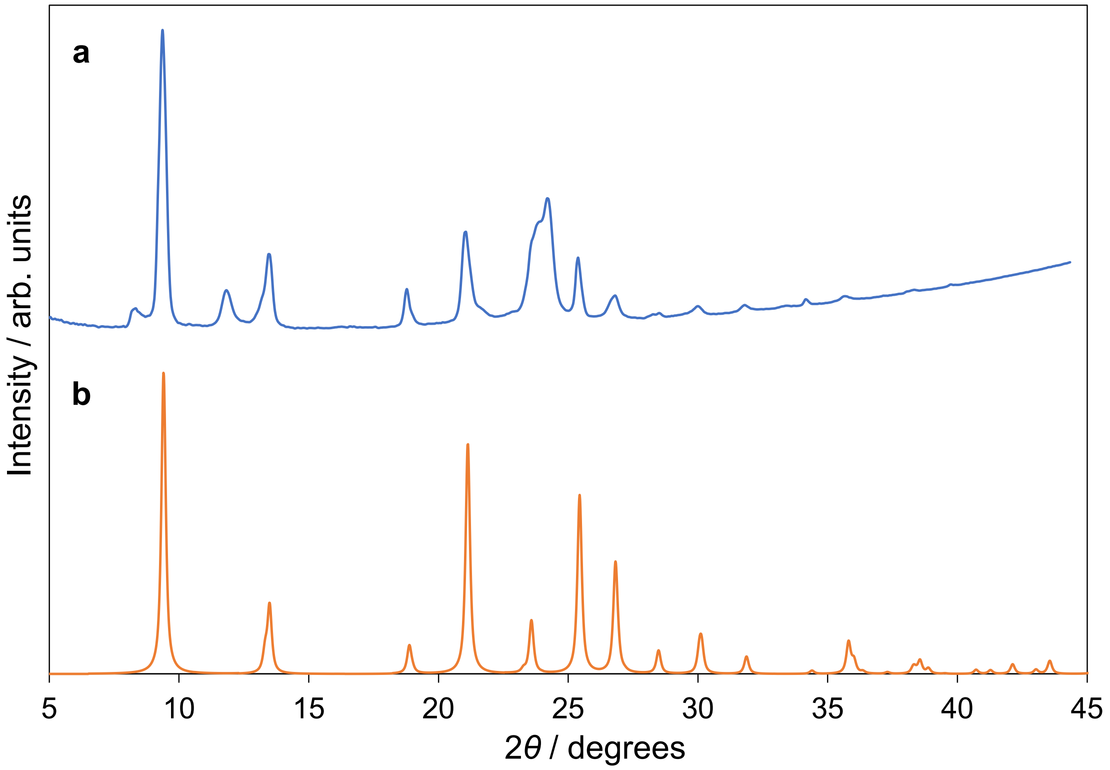

Supplementary Figure 5 | Powder XRD patterns of a bulk sample of 3D⊃benzene. **a**) Experimental pattern of a sample of 3D⊃benzene from which benzene had been removed by exposure to reduced pressure (400 Pa) for 27 hours at room temperature. **b**) Simulated pattern based on the data obtained from the single-crystal X-ray diffraction analysis of 3D⊃benzene[1].

2. X-ray crystallography and data

Single-crystal X-ray diffraction analyses were performed using a Rigaku XtaLAB P200 diffractometer with a Pilatus 200K detector and a multi-layer mirror-monochromated Mo-Kα radiation source (λ = 0.71075 Å) under a N_2 stream (93 K). Data collection, cell refinement, and data reduction were carried out using the CrystalClear-SM Expert 2.0 software[2] and the CrysAlisPro[3] software. The structures were solved by direct methods using the program SHELXT-2015[4] and refined by full-matrix least-squares methods on F^2 using SHELXL-2015[5]. The materials for publication were prepared with the software Olex2[6] and Yadokari-XG 2009[7]. Each crystal coated with Parabar 10312 (Hampton Research) was mounted on a micro-loop. CCDC 2074152 – 2074164 contain the supplementary crystallographic data for 1D-C, 1D-W, 1D-R, 2D-S, 2D-MS, 3D⊃THF, 3D⊃benzene, 3D⊃thiophene, 3D⊃selenophene, 3D⊃*p*-benzoquinone, 3D⊃THF·*p*-benzoquinone, 3D⊃thiophene·*p*-benzoquinone, and 3D⊃benzene·*p*-benzoquinone, respectively. These data can be obtained free of charge from The Cambridge Crystallographic Data Centre at www.ccdc.cam.ac.uk/data_request/cif.

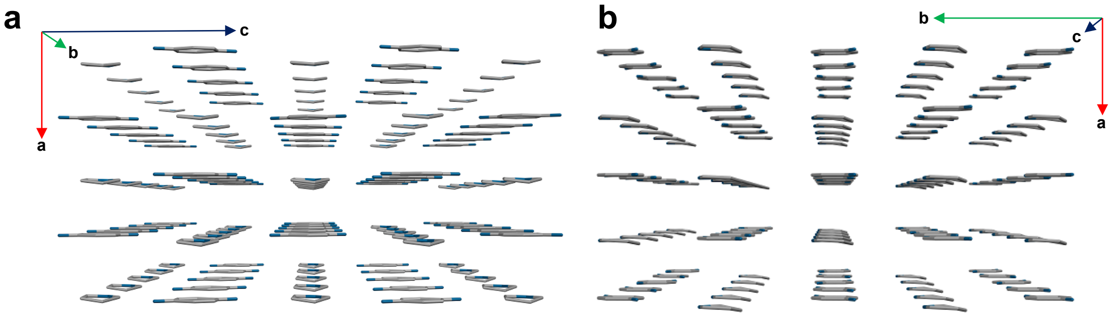

Supplementary Figure 6 | 3D crystal packing of guest molecules in 3D⊃THF·*p*-benzoquinone with 1 omitted for clarity. **a** View along the *b*-axis. **b** View along the *c*-axis.

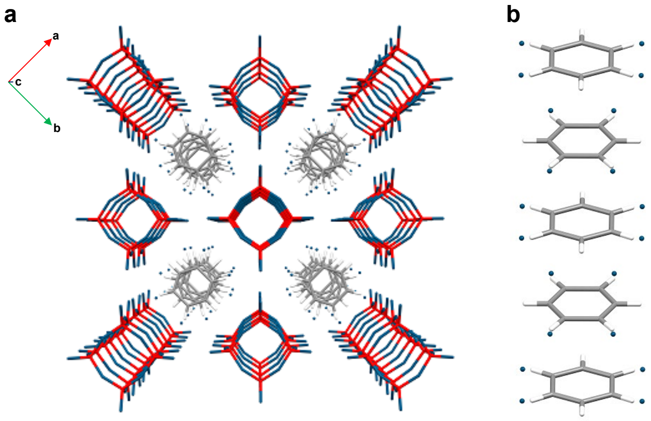

Supplementary Figure 7 | Crystal structure of 3D⊃benzene·*p*-benzoquinone determined by a single-crystal X-ray diffraction. Disordered benzene and *p*-benzoquinone molecules are shown. Color code: Si = red, O = blue, C = gray. The hydrogen atoms of the framework have been omitted for clarity. Crystal structure of 3D⊃benzene·*p*-benzoquinone showing **a**) 3D crystal packing; **b**) partial views of the stacked benzene and *p*-benzoquinone molecules to clarify the stacking.

Supplementary Table 1| Crystal data and structure refinement parameters for $[Si_8O_{12}][OH]_8\cdot 5DMAc$ (1D-C)

	1D-C	
Empirical formula	C40 H106 N10 O50 Si16	
Formula weight	1976.78	
Temperature	93 K	
Wavelength	0.71073 Å	
Crystal system	Triclinic	
Space group	P -1	
Unit cell dimensions	*a* = 10.4477(4) Å	α = 85.279(3)°
	b = 12.2738(4) Å	β = 81.090(3)°
	c = 17.9830(6) Å	γ = 71.375(3)°
Volume	2157.55(14) $Å^3$	
Z	1	
Density (calculated)	1.521 Mg/m^3	
Absorption coefficient	0.339 mm^{-1}	
F(000)	1040	
Crystal size	0.365 × 0.181 × 0.091 mm^3	
Theta range for data collection	2.231 to 28.819°	
Index ranges	-13≤*h*≤14, -16≤*k*≤15, -24≤*l*≤24	
Reflections collected	34403	
Independent reflections	10032 [*R*(int) = 0.0266]	
Completeness to θ = 25.242°	99.7%	
Refinement method	Full-matrix least-squares on F^2	
Data / restraints / parameters	10032 / 30 / 610	
Goodness-of-fit on F^2	1.030	
Final *R* indices [$I > 2\sigma(I)$]	R1 = 0.0371, wR2 = 0.0995	
R indices (all data)	R1 = 0.0472, wR2 = 0.1044	
Extinction coefficient	n/a	
Largest diff. peak and hole	0.978 and -0.315 e. $Å^{-3}$	

Supplementary Table 2| Crystal data and structure refinement parameters for $[Si_8O_{12}][OH]_8$·2DMAc·4THF (1D-W)

	1D-W	
Empirical formula	C24 H58 N2 O26 Si8	
Formula weight	1015.44	
Temperature	93 K	
Wavelength	0.71075 Å	
Crystal system	Triclinic	
Space group	P -1	
Unit cell dimensions	a = 9.2312(19) Å	α = 71.230(14)°
	b = 11.9320(17) Å	β = 68.288(8)°
	c = 12.2656(17) Å	γ = 67.329(8)°
Volume	1132.6(3) $Å^3$	
Z	1	
Density (calculated)	1.489 Mg/m^3	
Absorption coefficient	0.325 mm^{-1}	
F(000)	536	
Crystal size	0.12 × 0.11 × 0.06 mm^3	
Theta range for data collection	3.482 to 27.600°	
Index ranges	$-11\leq h\leq 11$, $-15\leq k\leq 10$, $-13\leq l\leq 15$	
Reflections collected	9719	
Independent reflections	4856 [R(int) = 0.1124]	
Completeness to θ = 25.242°	96.3%	
Refinement method	Full-matrix least-squares on F^2	
Data / restraints / parameters	4856 / 42 / 324	
Goodness-of-fit on F^2	0.976	
Final R indices [$I > 2\sigma(I)$]	R1 = 0.0591, wR2 = 0.1548	
R indices (all data)	R1 = 0.0953, wR2 = 0.1724	
Extinction coefficient	n/a	
Largest diff. peak and hole	0.870 and -0.765 e. $Å^{-3}$	

Supplementary Table 3| Crystal data and structure refinement parameters for $[Si_8O_{12}][OH]_8·4TMU$ (1D-R)

	1D-R	
Empirical formula	C20 H56 N8 O24 Si8	
Formula weight	1017.44	
Temperature	93 K	
Wavelength	0.71075 Å	
Crystal system	Monoclinic	
Space group	C 2/c	
Unit cell dimensions	a = 24.691(5) Å	α = 90°
	b = 7.5453(16) Å	β = 92.492(7)°
	c = 24.954(6) Å	γ = 90°
Volume	4644.6(18) $Å^3$	
Z	4	
Density (calculated)	1.455 Mg/m^3	
Absorption coefficient	0.317 mm^{-1}	
F(000)	2144	
Crystal size	0.266 × 0.173 × 0130 mm^3	
Theta range for data collection	3.244 to 27.496°	
Index ranges	$-32 \le h \le 31$, $-9 \le k \le 8$, $-29 \le l \le 32$	
Reflections collected	22030	
Independent reflections	5017 [R(int) = 0.0223]	
Completeness to θ = 25.242°	96.3%	
Refinement method	Full-matrix least-squares on F^2	
Data / restraints / parameters	5017 / 0 / 324	
Goodness-of-fit on F^2	1.138	
Final R indices [$I > 2\sigma(I)$]	R1 = 0.0431, wR2 = 0.1024	
R indices (all data)	R1 = 0.0522, wR2 = 0.1069	
Extinction coefficient	n/a	
Largest diff. peak and hole	0.345 and -0.311 e. $Å^{-3}$	

Supplementary Table 4| Crystal data and structure refinement parameters for $[Si_8O_{12}][OH]_8 \cdot 2Et_2O$ (2D-S)

	2D-S	
Empirical formula	C8 H28 O22 Si8	
Formula weight	701.02	
Temperature	93 K	
Wavelength	0.71073 Å	
Crystal system	Monoclinic	
Space group	P 2_1/n	
Unit cell dimensions	a = 11.2551(5) Å	α = 90°
	b = 7.5487(3) Å	β = 99.620(4)°
	c = 16.9443(6) Å	γ = 90°
Volume	1419.37(10) $Å^3$	
Z	2	
Density (calculated)	1.640 Mg/m^3	
Absorption coefficient	0.466 mm^{-1}	
F(000)	728	
Crystal size	0.13 × 0.11 × 0.04 mm^3	
Theta range for data collection	2.026 to 30.763°	
Index ranges	$-15 \le h \le 16$, $-10 \le k \le 10$, $-24 \le l \le 23$	
Reflections collected	10804	
Independent reflections	10804	
Completeness to θ = 25.242°	99.1%	
Refinement method	Full-matrix least-squares on F^2	
Data / restraints / parameters	10804 / 0 / 179	
Goodness-of-fit on F^2	0.944	
Final R indices [$I > 2\sigma(I)$]	R1 = 0.0519, wR2 = 0.1294	
R indices (all data)	R1 = 0.0775, wR2 = 0.1322	
Extinction coefficient	n/a	
Largest diff. peak and hole	0.741 and -0.477 e. $Å^{-3}$	

Supplementary Table 5| Crystal data and structure refinement parameters for $[Si_8O_{12}][OH]_8{\cdot}6DMAc$ (2D-MS)

	2D-MS	
Empirical formula	C24 H62 N6 O26 Si8	
Formula weight	1075.51	
Temperature	93 K	
Wavelength	0.71073 Å	
Crystal system	Monoclinic	
Space group	C 2/c	
Unit cell dimensions	a = 21.0217(6) Å	α = 90°
	b = 16.9100(5) Å	β = 96.218(3)°
	c = 27.0242(8) Å	γ = 90°
Volume	9550.0(5) $Å^3$	
Z	8	
Density (calculated)	1.496 Mg/m^3	
Absorption coefficient	0.315 mm^{-1}	
F(000)	4544	
Crystal size	0.14 × 0.05 × 0.04 mm^3	
Theta range for data collection	2.409 to 30.680°	
Index ranges	$-30 \le h \le 29$, $-23 \le k \le 24$, $-37 \le l \le 37$	
Reflections collected	64977	
Independent reflections	13460 [R(int) = 0.0516]	
Completeness to θ = 25.242°	99.7%	
Refinement method	Full-matrix least-squares on F^2	
Data / restraints / parameters	13458 / 172 / 718	
Goodness-of-fit on F^2	1.054	
Final R indices [$I > 2\sigma(I)$]	R1 = 0.0584, wR2 = 0.1327	
R indices (all data)	R1 = 0.0998, wR2 = 0.1492	
Extinction coefficient	n/a	
Largest diff. peak and hole	1.228 and -0.933 e. $Å^{-3}$	

Supplementary Table 6| Crystal data and structure refinement parameters for $[Si_8O_{12}][OH]_8 \cdot n$THF (3D⊃THF)[a]

	3D⊃THF	
Empirical formula	Si8O20H8	
Formula weight	552.78	
Temperature	93 K	
Wavelength	0.71073 Å	
Crystal system	Tetragonal	
Space group	P 4/m n c	
Unit cell dimensions	a = 13.2096(10) Å	α = 90°
	b = 13.2096(10) Å	β = 90°
	c = 7.5389(6) Å	γ = 90°
Volume	1315.5(2) $Å^3$	
Z	2	
Density (calculated)	1.396 Mg/m^3	
Absorption coefficient	0.477 mm^{-1}	
F(000)	560	
Crystal size	0.21 × 0.20 × 0.05 mm^3	
Theta range for data collection	3.084 to 30.753°	
Index ranges	$-12 \le h \le 18$, $-18 \le k \le 18$, $-9 \le l \le 10$	
Reflections collected	17057	
Independent reflections	1041 [R(int) = 0.1235]	
Completeness to θ = 25.242°	99.05%	
Refinement method	Full-matrix least-squares on F^2	
Data / restraints / parameters	1041 / 0 / 34	
Goodness-of-fit on F^2	1.885	
Final R indices [$I > 2\sigma(I)$]	R1 = 0.1658, wR2 = 0.4630	
R indices (all data)	R1 = 0.1905, wR2 = 0.4739	
Extinction coefficient	n/a	
Largest diff. peak and hole	2.261 and -0.789 e. $Å^{-3}$	

a: In the crystal structure, THF molecules were highly disordered and couldn't be properly modelled.

Supplementary Table 7| Crystal data and structure refinement parameters for $[Si_8O_{12}][OH]_8\cdot 2(C_6H_6)$ (3D⊃benzene)

	3D⊃benzene	
Empirical formula	C12 H20 O20 Si8	
Formula weight	709.00	
Temperature	93 K	
Wavelength	0.71075 Å	
Crystal system	Tetragonal	
Space group	I 4/m m m	
Unit cell dimensions	a = 13.297(4) Å	α = 90°
	b = 13.297(4) Å	β = 90°
	c = 7.546(3) Å	γ = 90°
Volume	1334.2(10) $Å^3$	
Z	2	
Density (calculated)	1.765 Mg/m^3	
Absorption coefficient	0.492 mm^{-1}	
F(000)	728	
Crystal size	0.18 × 0.04 × 0.04 mm^3	
Theta range for data collection	3.104 to 27.600°	
Index ranges	$-11\leq h\leq 11$, $-15\leq k\leq 10$, $-13\leq l\leq 15$	
Reflections collected	8686	
Independent reflections	470 [R(int) = 0.0287]	
Completeness to θ = 25.242°	99.5%	
Refinement method	Full-matrix least-squares on F^2	
Data / restraints / parameters	470 / 4 / 33	
Goodness-of-fit on F^2	1.148	
Final R indices [$I > 2\sigma(I)$]	R1 = 0.0383, wR2 = 0.1210	
R indices (all data)	R1 = 0.0413, wR2 = 0.1234	
Extinction coefficient	n/a	
Largest diff. peak and hole	0.491 and -0.479 e. $Å^{-3}$	

Supplementary Table 8| Crystal data and structure refinement parameters for $[Si_8O_{12}][OH]_8\cdot 2$(thiophene) (3D⊃thiophene)

	3D⊃thiophene	
Empirical formula	C8 H16 O20 S2 Si8	
Formula weight	721.05	
Temperature	93 K	
Wavelength	0.71073 Å	
Crystal system	Tetragonal	
Space group	I 4/m m m	
Unit cell dimensions	a = 13.2909(2) Å	$\alpha = 90°$
	b = 13.2909(2) Å	$\beta = 90°$
	c = 7.5453(2) Å	$\gamma = 90°$
Volume	1332.86(5) $Å^3$	
Z	2	
Density (calculated)	1.797 Mg/m^3	
Absorption coefficient	0.645 mm^{-1}	
F(000)	736	
Crystal size	0.25 × 0.03 × 0.03 mm^3	
Theta range for data collection	3.065 to 30.498°	
Index ranges	$-18 \le h \le 18$, $-16 \le k \le 18$, $-10 \le l \le 10$	
Reflections collected	9530	
Independent reflections	598 [R(int) = 0.0290]	
Completeness to θ = 25.242°	99.7%	
Refinement method	Full-matrix least-squares on F^2	
Data / restraints / parameters	598 / 70 / 64	
Goodness-of-fit on F^2	1.181	
Final R indices [$I > 2\sigma(I)$]	R1 = 0.0412, wR2 = 0.1364	
R indices (all data)	R1 = 0.0431, wR2 = 0.1387	
Extinction coefficient	n/a	
Largest diff. peak and hole	0.614 and -0.460 e. $Å^{-3}$	

Supplementary Table 9| Crystal data and structure refinement parameters for $[Si_8O_{12}][OH]_8 \cdot 2$(selenophene) (3D⊃selenophene)

	3D⊃selenophene	
Empirical formula	C8 H16 O20 Se2 Si8	
Formula weight	814.85	
Temperature	93 K	
Wavelength	0.71075 Å	
Crystal system	Tetragonal	
Space group	I 4/m m m	
Unit cell dimensions	*a* = 13.2851(7) Å	α = 90°
	b = 13.2851(7) Å	β = 90°
	c = 7.5409(6) Å	γ = 90°
Volume	1330.92(18) $Å^3$	
Z	2	
Density (calculated)	2.033 Mg/m^3	
Absorption coefficient	3.224 mm^{-1}	
F(000)	808	
Crystal size	0.17 × 0.03 × 0.03 mm^3	
Theta range for data collection	2.168 to 30.623°	
Index ranges	$-18 \le h \le 18$, $-18 \le k \le 18$, $-10 \le l \le 10$	
Reflections collected	9482	
Independent reflections	609 [*R*(int) = 0.0426]	
Completeness to θ = 25.242°	100.0%	
Refinement method	Full-matrix least-squares on F^2	
Data / restraints / parameters	609 / 98 / 66	
Goodness-of-fit on F^2	1.171	
Final *R* indices [$I > 2\sigma(I)$]	R1 = 0.0725, wR2 = 0.2284	
R indices (all data)	R1 = 0.0894, wR2 = 0.2576	
Extinction coefficient	n/a	
Largest diff. peak and hole	1.165 and -1.246 e. $Å^{-3}$	

Supplementary Table 10| Crystal data and structure refinement parameters for $[Si_8O_{12}][OH]_8 \cdot 2$(*p*-benzoquinone) (3D⊃*p*-benzoquinone)

	3D⊃*p*-benzoquinone	
Empirical formula	C12 H16 O24 Si8	
Formula weight	768.97	
Temperature	93 K	
Wavelength	0.71075 Å	
Crystal system	Tetragonal	
Space group	P 4/m n c	
Unit cell dimensions	*a* = 13.413(2) Å	α = 90°
	b = 13.413(2) Å	β = 90°
	c = 7.5121(17) Å	γ = 90°
Volume	1351.5(5) Å^3	
Z	2	
Density (calculated)	1.890 Mg/m^3	
Absorption coefficient	0.504 mm^{-1}	
F(000)	784	
Crystal size	0.08 × 0.07 × 0.03 mm^3	
Theta range for data collection	3.037 to 27.476°	
Index ranges	$-16 \le h \le 14$, $-17 \le k \le 17$, $-9 \le l \le 7$	
Reflections collected	15936	
Independent reflections	837 [*R*(int) = 0.0326]	
Completeness to θ = 25.242°	99.7%	
Refinement method	Full-matrix least-squares on F^2	
Data / restraints / parameters	837 / 0 / 59	
Goodness-of-fit on F^2	1.137	
Final *R* indices [$I > 2\sigma(I)$]	R1 = 0.0332, wR2 = 0.0944	
R indices (all data)	R1 = 0.0371, wR2 = 0.0979	
Extinction coefficient	n/a	
Largest diff. peak and hole	0.318 and -0.693 e. Å^{-3}	

Supplementary Table 11| Crystal data and structure refinement parameters for $[Si_8O_{12}][OH]_8$·THF·*p*-benzoquinone (3D⊃THF·*p*-benzoquinone)

	3D⊃THF·*p*-benzoquinone	
Empirical formula	C10 H20 O23 Si8	
Formula weight	732.98	
Temperature	93 K	
Wavelength	0.71073 Å	
Crystal system	Orthorhombic	
Space group	I m m m	
Unit cell dimensions	*a* = 7.5287(3) Å	α = 90°
	b = 13.1402(6) Å	β = 90°
	c = 13.5084(6) Å	γ = 90°
Volume	1336.37(10) Å^3	
Z	2	
Density (calculated)	1.822 Mg/m^3	
Absorption coefficient	0.502 mm^{-1}	
F(000)	752	
Crystal size	0.12 × 0.07 × 0.03 mm^3	
Theta range for data collection	3.016 to 30.633°	
Index ranges	-10≤*h*≤10, -17≤*k*≤17, -18≤*l*≤19	
Reflections collected	9528	
Independent reflections	1121 [*R*(int) = 0.0508]	
Completeness to θ = 25.242°	99.9%	
Refinement method	Full-matrix least-squares on F^2	
Data / restraints / parameters	1121 / 171 / 104	
Goodness-of-fit on F^2	1.106	
Final *R* indices [$I > 2\sigma(I)$]	R1 = 0.0573, wR2 = 0.1659	
R indices (all data)	R1 = 0.0708, wR2 = 0.1743	
Extinction coefficient	n/a	
Largest diff. peak and hole	0.981 and -1.225 e. Å^{-3}	

Supplementary Table 12| Crystal data and structure refinement parameters for $[Si_8O_{12}][OH]_8$·thiophene·*p*-benzoquinone(3D⊃thiophene·*p*-benzoquinone)

	3D⊃thiophene·*p*-benzoquinone	
Empirical formula	C10 H16 O22 S Si8	
Formula weight	745.01	
Temperature	93 K	
Wavelength	0.71075 Å	
Crystal system	Orthorhombic	
Space group	I m m m	
Unit cell dimensions	a = 7.536(3) Å	α = 90°
	b = 13.113(4) Å	β = 90°
	c = 13.526(5) Å	γ = 90°
Volume	1336.6(8) $Å^3$	
Z	2	
Density (calculated)	1.851 Mg/m^3	
Absorption coefficient	0.576 mm^{-1}	
F(000)	760	
Crystal size	0.10 × 0.03 × 0.02 mm^3	
Theta range for data collection	3.012 to 27.460°	
Index ranges	$-9 \le h \le 8$, $-16 \le k \le 16$, $-16 \le l \le 16$	
Reflections collected	8662	
Independent reflections	866 [R(int) = 0.0685]	
Completeness to θ = 25.242°	98.6%	
Refinement method	Full-matrix least-squares on F^2	
Data / restraints / parameters	866 / 168 / 112	
Goodness-of-fit on F^2	1.068	
Final R indices [$I > 2\sigma(I)$]	R1 = 0.0551, wR2 = 0.1510	
R indices (all data)	R1 = 0.0683, wR2 = 0.1615	
Extinction coefficient	n/a	
Largest diff. peak and hole	0.629 and -0.982 e. $Å^{-3}$	

Supplementary Table 13| Crystal data and structure refinement parameters for $[Si_8O_{12}][OH]_8$·benzene·*p*-benzoquinone (3D⊃benzene·*p*-benzoquinone)

	3D⊃benzene·*p*-benzoquinone	
Empirical formula	C12 H18.75 O21.25 Si8	
Formula weight	727.66	
Temperature	93 K	
Wavelength	0.71073 Å	
Crystal system	Tetragonal	
Space group	I 4/m m m	
Unit cell dimensions	a = 13.3714(14) Å	α = 90°
	b = 13.3714(14) Å	β = 90°
	c = 7.5271(10) Å	γ = 90°
Volume	1345.8(3) $Å^3$	
Z	2	
Density (calculated)	1.796 Mg/m^3	
Absorption coefficient	0.493 mm^{-1}	
F(000)	745	
Crystal size	0.05 × 0.04 × 0.02 mm^3	
Theta range for data collection	3.047 to 30.632°	
Index ranges	$-17 \le h \le 18$, $-18 \le k \le 18$, $-10 \le l \le 10$	
Reflections collected	9609	
Independent reflections	612 [R(int) = 0.2134]	
Completeness to θ = 25.242°	99.7%	
Refinement method	Full-matrix least-squares on F^2	
Data / restraints / parameters	612 / 9 / 41	
Goodness-of-fit on F^2	1.117	
Final R indices [$I > 2\sigma(I)$]	R1 = 0.0739, wR2 = 0.1845	
R indices (all data)	R1 = 0.1067, wR2 = 0.2056	
Extinction coefficient	n/a	
Largest diff. peak and hole	0.972 and -0.756 e. $Å^{-3}$	

Supplementary Table 14| Closest distances between the oxygen atoms in $[Si_8O_{12}][OH]_8$ and the atoms of the guest molecules in crystals of 3D⊃guest

crystal	O(host) – atom(guest)	Distances (Å)
3D⊃benzene	O_{SiOSi} – C	3.734(9)
	O_{SiOH} – C	3.922(6)
3D⊃thiophene	O_{SiOSi} – S	3.649(19)
	O_{SiOH} – S	3.92(2)
	O_{SiOSi} – C	3.60(5)
	O_{SiOH} – C	3.57(3)
3D⊃selenophene	O_{SiOSi} – Se	3.75(3)
	O_{SiOH} – Se	3.91(2)
	O_{SiOSi} – C	3.99(8)
	O_{SiOH} – C	3.68(9)
3D⊃*p*-benzoquinone	O_{SiOSi} – O	2.850(5)
	O_{SiOH} – O	3.470(5)
	O_{SiOSi} – C	3.705(6)
	O_{SiOH} – C	3.678(5)

Supplementary Table 15| CCDC codes for the crystal structure of compounds reported herein

Figure	Compound	CCDC Deposition Number
Figure 1a–c	1D-C	2074152
Figure 1d–f	1D-W	2074153
Figure 1j–l	1D-R	2074154
Figure 2c-e	2D-S	2074155
Figure 2f–i	2D-MS	2074156
Figure 4d-i	3D⊃benzene	2074158
Figure 6a-c	3D⊃thiophene	2074159
Figure 6d-f	3D⊃selenophene	2074160
Figure 7a–c	3D⊃*p*-benzoquinone	2074161
Figure 7d,e, Supplementary Figure 6a,b	3D⊃THF·*p*-benzoquinone	2074162
Figure 7f–i	3D⊃thiophene·*p*-benzoquinone	2074163
Supplementary Figure 2a-c	3D⊃THF	2074157
Supplementary Figure 7a,b	3D⊃benzene·*p*-benzoquinone	2074164

3. Supplementary References

1 Mercury 2020.2: Macrae, C. F. *et al. Mercury 4.0*: from visualization to analysis, design and prediction. *J. Appl. Cryst.* **53**, 226–235 (2020).

2 *CrystalClear-SM Expert*: Rigaku Corporation, Tokyo, Japan, 2011.

3 *CrysAlisPro:* Rigaku Corporation, Tokyo, Japan, Oxford, UK, 2015.

4 *SHELXT–2015*: G. M. Sheldrick, Program for the Solution of Crystal Structures; *Acta Crystallogr. A* 2015, **71**, 3–8.

5 *SHELXL–2015*: G. M. Sheldrick, Program for the Refinement of Crystal Structures; *Acta Crystallogr. C* 2015, **71**, 3–8.

6 O. V. Dolomanov, L. J. Bourhis, R.J. Gildea, J.A.K. Howard, H. Puschmann, *OLEX2: A complete structure solution, refinement and analysis program*. *J. Appl. Cryst.* 2009, **42**, 339-341.

7 a) *Yadokari–XG*: K. Wakita, Software for crystal Structure Analyses, **2001**. b) *Yadokari–XG 2009*: C. Kabuto, S. Akine, T. Nemoto, E. Kwon, Release of Software for Crystal Structure Analyses; *J. Cryst. Soc. Jpn.* **2009**, *51*, 218–224.
